# PROMIS® Health Organization (PHO) 2021 Conference Abstracts

**DOI:** 10.1186/s41687-021-00349-3

**Published:** 2021-10-01

## 1* Intentionally omitted

### P2 Patient reported measures—driving system transformation through measuring what matters to our patients in New South Wales

#### Melissa Tinsley

##### Agency for Clinical Innovation, Sydney, Australia

###### Correspondence: Melissa Tinsley

melissa.tinsley@health.nsw.gov.au

*Journal of Patient-Reported Outcomes* 5(1): P2

**Objective**: NSW health has embarked on a Value Based Healthcare approach. A key enabler of this is the Patient Reported Measures (PRM) program to achieve the quadruple aim.

**Methods:** The PRM program is a whole of health system approach across the continuum of care. Using PROMIS 29 as the generic HRQoL measure alongside condition specific measures as a survey set. Surveys are completed electronically (email/SMS or tablet in face to face), data is available in real-time for clinicians and care teams to review for shared decision making, care planning, treatment and interventions.

The PRM Program has bought people, process and technology together for a robust change management approach and transforming culture and behaviour to become truly person centred.

This presentation will highlight the key success factors and how we overcame challenges to achieve large system transformation, and describing how the data is used at the individual, service and system level.

**Results:** The PRM Program, including our technology platform continues to be implemented across NSW with high engagement and success. The data collected from PROMIS 29 is used at the individual, service and system level.

Leveraging off the success of our initial implementation we are continuing to co-design and expand our program, including culturally and linguistically diverse patient populations. The next phase of our program will include the translated PROMIS 29 surveys so consumers and carers can answer their surveys in their preferred language for greater inclusivity.

**Conclusions:** In conclusion, the NSW Health PRM Program, is the largest program collecting and using PRMs, including PROMIS 29 in Australia; and perhaps even broader—having far reaching coverage across patient cohorts/conditions and settings.

The e-enablement of technology and presenting data back in a useful, meaningful and relevant context for healthcare teams in real-time has been one of the main success factors of the program.

## 3* Intentionally omitted

### P4 Relationship between depression and social function among patients with cerebral infarction based on patient-reported outcomes

#### Xiaoping Song, Zhiren Sheng, Kaili Sun, Chunbo Liu, Hongdi Zhou, Jianli Hu, Qingwen Su

##### The Affiliated Hospital of Medical School, Ningbo, Ningbo, China

###### Correspondence: Xiaoping Song

sunkaili2015@163.com

*Journal of Patient-Reported Outcomes* 5(1): P4.

**Objective:** To explore the relationship between depression and social function, and mediating effect of cognitive function on the relationship between the depression and social function among patients with cerebral infarction.

**Methods:** A convenience sampling method was used to investigate 156 cerebral infarction patients undergoing conservative treatment 8 weeks after discharge. The general information questionnaire, PROMIS-depression short form, PROMIS-cognitive function short form, PROMIS-ability to participate in social roles and activities short form were adopted from January 2020 to January 2021.

**Results:** For all the participants, the mean scores of PROMIS-depression short form was (14.3 ± 6.6), the mean score of PROMIS-cognitive function short form was (15.6 ± 4.3), and the mean score of PROMIS-ability to participate in social roles and activities short form was (14.4 ± 5.8). The results of correlation analysis revealed that the score of PROMIS-depression short form were negatively correlated with score of PROMIS-cognitive function short form ( P < 0.01) and score of PROMIS-ability to participate in social roles and activities short form (P < 0.01). The score of PROMIS-cognitive function short form were positively correlated with score of PROMIS-ability to participate in social roles and activities short form (P < 0.01). The analysis results by Bias Corrected Bootstrap showed that cognitive function played a partial mediation effect between the depression and the ability to participate in social roles and activities [(a × b = − 0.165, 95% CI = − 0.294 ~ − 0.068)].

**Conclusions:** Patients with cerebral infarction had poor ability to participate in social roles and activities. Depression can directly influence the ability to participate in social roles and activities and indirectly influence the ability to participate in social roles and activities through cognitive function.

### O5 A patient-centric PROMIS care continuum: tools to encourage patient and provider use of PROMIS data

#### Stacy Schmitt^1^, Martha Springsted^1^, Sasha Ford^1^, William Mauck^2^, Nafisseh Warner^2^, Kevin Hardesty^3^, Christin Tiegs-Heiden^4^, Timothy Maus^4^, Mark Nyman^5^

##### ^1^Mayo Clinic Multidisciplinary Spine Center, Rochester, MN,USA. ^2^Mayo Clinic Department of Anesthesiology, Rochester, MN, USA. ^3^Mayo Clinic Department of Family Practice, Rochester, MN, USA. ^4^Mayo Clinic Department of Radiology, Rochester, MN, USA. ^5^Mayo Clinic Department of General Internal M, Rochester, MN, USA

###### Correspondence: Stacy Schmitt

schmitt.stacy@mayo.edu

*Journal of Patient-Reported Outcomes* 5(1): O5.

**Objective:** Building and promoting an individualized program is essential for the collection and clinical use of patient reported outcomes (PROs). The objective is to describe the identification and deployment of individualized collection tools the Mayo Clinic Spine Care Continuum administers to achieve PRO collection goals, and the visualization tools that maximize clinical utility for providers and patients throughout the five-year PRO cadence.

**Methods:** Collaborative efforts between 14 Mayo spine care provider specialties (> 1,000 providers) identified programmatic deployment criteria to collect PROs on meaningful and complete patient populations. Specialty stakeholder input was used to build patient-centric and provider-friendly visualizations within the Epic EHR.

**Results:** Consensus was achieved among Spine provider specialties on appropriate deployment criteria (i.e., triage protocols, diagnoses, visit types, procedure codes and surgical preference card IDs) for baseline PRO collection, series cadence, and PROMIS CAT domains. Standardization of criteria across all specialties was sought, while still allowing necessary customization for procedural specialties to encompass pre-authorization and regulatory needs. Metrics and goals for baseline and subsequent cadence collection were identified: PRO collection modes integrated within the Epic EHR targeted a baseline collection of 95%; patient-centric collection modes were developed to achieve a subsequent cadence collection of 80%. Customized provider visualizations within the Epic EHR utilized Synopsis and Print Groups, noting anchor events that include care progression, procedural and surgical care interventions. Epic data extracted into a Tableau Dashboard displays collection rates by location, specialty, provider and patient demographic. Collection rates are broadly distributed quarterly through newsletters, education and awareness encounters, and specialty-specific meetings. Ongoing education and awareness interactions are essential practice support tools to ensure understanding and execution of collection methodology and optimal use of customized, patient-centric PROMIS data.

**Conclusions:** A patient-centric, programmatic deployment system was created within the Epic EHR based on individualized criteria within meaningful populations throughout the Spine Care Continuum. Multiple response modes are necessary to meet program metrics and will be presented at multiple cadence intervals. Additionally, customized visualizations for individualized patient/provider interactions within the Epic EHR will be displayed. Analytics used to measure collection rates and assist their optimization will be presented and discussed.

### P6 Psychometric evaluation of the PROMIS Social Function Short Forms in Chinese patients with breast cancer

#### Tingting Cai, Changrong Yuan, Qingmei Huang, Fulei Wu, Haozhi Xia

##### School of Nursing, Fudan University, Shanghai, China

###### Correspondence: Tingting Cai

caitingtingguo@163.com

*Journal of Patient-Reported Outcomes* 5(1): P6.

**Objective:** A valid assessment of social function is important for the follow-up of patients with breast cancer. This study aimed to carry out a cross-cultural adaptation and analyzed the psychometric properties of the PROMIS Social Function Short Forms in a sample of Chinese patients with breast cancer.

**Methods:** After a standardized transcultural adaptation process, a psychometric evaluation was performed regarding the reliability and construct validity of the scales. Using convenience sampling, eligible patients with breast cancer from tertiary hospitals in China were enrolled from January 2018 to July 2020. Participants completed the sociodemographic information questionnaire, the PROMIS Social Function Short Forms, the Functional Assessment of Cancer Therapy-Breast, the PROMIS Emotional Support Short Form, and the PROMIS Anxiety Short Form.

**Results:** The sample was composed of 633 patients, with an average age of 48.1 years. The PROMIS Social Function Short Forms showed adequate internal consistency and absence of floor or ceiling effects. Regarding construct validity, the results of confirmatory factor analysis supported the original structure of the PROMIS Social Function Short Forms. Correlations with the PROMIS Emotional Support Short Form scores, the Functional Assessment of Cancer Therapy-Breast scores and the PROMIS Anxiety Short Form scores showed evidence of satisfactory convergent and discriminant validity. Additionally, the measures were invariant across patients with different age and education.

**Conclusions:** The findings suggested that the Chinese version of the PROMIS Social Function Short Forms had acceptable reliability and validity. Additional psychometric evaluation is further needed to draw firm conclusions.

### P7 Social relationships and its predictive factors in Chinese patients with breast cancer: a cross-sectional study

#### Changrong Yuan, Tingting Cai, Qingmei Huang, Fulei Wu, Haozhi Xia

##### School of Nursing, Fudan University, Shanghai, China

###### Correspondence: Changrong Yuan

yuancr@fudan.edu.cn

*Journal of Patient-Reported Outcomes* 5(1): P7.

**Objective:** Social relationships have been viewed as important to help patients to cope with their diseases. However, social relationships of patients with breast cancer still remain unaddressed in clinical settings. The aim of this study was to evaluate emotional, informational, and instrumental support needs in Chinese patients with breast cancer, and explore the predictors.

**Methods:** A cross-sectional research design was adopted. Using convenience sampling, eligible patients with breast cancer from tertiary hospitals in China were recruited and completed the sociodemographic information questionnaire, the PROMIS Social Relationships Short Form, the PROMIS Anxiety Short Form, and the PROMIS Depression Short Form.

**Results:** A total of 461 patients were investigated, with an average age of 50.9 years. T score of informational support was lower than the average level while emotional and informational support were in the average level. Marriage status, childbearing history, lifestyle, current employment, anxiety and depression level were found to be related to the emotional, informational, and instrumental support scores. Regression analyses revealed that emotional support, instrumental support, marriage status, current employment, anxiety and depression outcomes were predictors for informational support.

**Conclusions:** Informational support should be specially assessed and promoted in patients with breast cancer. Marriage status, childbearing history, lifestyle, current employment, anxiety and depression symptoms should be considered and evaluated when conducting interventions to promote emotional, informational, and instrumental support in patients with breast cancer.

### O8 Clinical sensitivity of PROMIS physical and mental quality of life domains to radiation therapy

#### Todd DeWees^1^, Feven Abraha^2^, Kimberly Corbin^2^, Paul Brown^2^, Chris Hallemeier^2^, Brian Davis^2^, Ivy Petersen^2^, James Martenson^2^, Safia Ahmed^2^, Kenneth Olivier^2^, Tamara Vern-Gross^3^, William Wong^3^, Sujay Vora^3^, Jonathan Ashman^3^, Steven Schild^3^, Daniel Trifiletti^4^, Carlos Vargas^3^, Daniel Ma^2^

##### ^1^Mayo Clinic, Scottsdale, AZ, USA. ^2^Mayo Clinic, Rochester, MN, USA. ^3^Mayo Clinic, Phoenix, AZ, USA. ^4^Mayo Clinic, Jacksonville, FL, USA.

###### Correspondence: Todd DeWees

dewees.todd@mayo.edu

*Journal of Patient-Reported Outcomes* 5(1): O8.

**Objective:** Patient-Reported Outcomes Measurement Information System 10 (PROMIS-10) is utilized for quantifying changes in patient quality of life (QOL) before and after medical intervention. The sensitivity of the PROMIS-10 instrument to radiotherapy (RT) was evaluated, with specific interest in meaningful clinically important differences (MCID) in the Global Physical and Mental domains.

**Methods:** A prospective registry to capture provider- and patient-reported outcomes was instituted in a large, multi-site radiation oncology practice. Patients treated with RT between 2013 and 2020 were administered PROMIS-10 questionnaires at baseline, end-of-treatment, 3, 6, 12 months, then annually. A change in PROMIS scores from pre-RT to end-of-treatment (EOT) by at least 4 points was considered MCID. ANOVA models estimated the mean differences between pre-RT and EOT and between treatment groups. Tukey’s adjustment for multiple comparisons was utilized and 95% confidence intervals (CI) were computed for all estimates.

**Results:** 6,456 patients were eligible; 54% were males with an average age of 61.7 years old (range:6–93 years). Patient disease sites were: GU(28%), Breast(26.2%), H&N(11%), CNS(8.5%), Thoracic(5.2%), Pancreas-Biliary(4.7%), Soft-Tissue/Bone(4.6%), Esophagus-Gastric(3.8%), GYN(2.6%), Heme/Lymph(2.3%), Colorectal-Anus(1.7%), and Skin/Melanoma(1.5%).

Global Physical Health significantly decreased for Colorectal-Anus (5.06; 95% CI: 3.8–6.3), Esophagus-Gastric (5.05; 95% CI: 4.2–5.9), and H&N(4.72; 95% CI: 4.2–5.2) patients. All other disease sites were not significantly different with mean decreases ranging from 0.5 to 2.3 points.

In contrast, Global Mental Health demonstrated impact in mental health for Colorectal-Anus (1.77; 95% CI: 0.5–3.0), Esophagus-Gastric(1.93; 95% CI: 1.1–2.7), and H&N(2.95; 95% CI: 2.5–3.4) patients; however, none reached MCID. Mean scores for ranged from a 0.31-point improvement to 2.95-point decline.

**Conclusions:** This prospective registry demonstrates that the PROMIS-10 Global Physical Health is sensitive in identifying clinically meaningful changes due to RT in patients with typically higher burden, chemo-RT treated tumors for Colorectal-Anus, Esophagus-Gastric, and H&N cancers. However, it did not detect meaningful changes for other cancer patients receiving RT, demonstrating possible need for investigation into other QOL measures while ensuring a balance between clinical utility and patient survey burden.

### P9 Mental health is associated with divergence of symptom severity and fracture severity

#### Prakash Jayakumar^1,2^, Aresh Al Salman^1^, Romil Shah^1^, Jacob Thomas^1^, David Ring^1^, Tom Crijns^1^, Stephen Gwilym^2^

##### ^1^The University of Texas of Austin, Dell Medical School, Austin, TX, USA. ^2^Oxford University, Oxford, United Kingdom

###### Correspondence: Prakash Jayakumar

pjx007@gmail.com

*Journal of Patient-Reported Outcomes* 5(1): P9.

**Objective**: There is notable variation in pain intensity and magnitude of activity intolerance after fracture. The relative degree of variation accounted for by mental health may be different for different degrees of fracture severity, and this may change as recovery progresses. Using a dataset of mental health and patient reported outcome measures (PROMs) from a large longitudinal cohort of people recovering from an upper extremity fracture, the objective of this study was to measure the association of both pain intensity and magnitude of activity intolerance with fracture severity in the presence of greater symptoms of depression and catastrophic thinking at both early and later stages of recovery.

**Methods**: We analyzed data from a longitudinal cohort of 731 patients recovering from a shoulder, elbow or wrist fracture. Patients completed a visual analogue measure of pain intensity and questionnaires measuring upper extremity activity intolerance (Quick Disabilities of Arm, Shoulder and Hand, QuickDASH), symptoms of depression (Patient Reported Outcome Measurement Information System (PROMIS) Depression), and catastrophic thinking (Pain Catastrophization Scale, PCS) between 2 and 4 weeks after injury and between 6 and 9 months after injury. At both time points, we measured the association of mental health with variation in (the statistical term being “moderation of”) pain intensity and magnitude of activity intolerance experienced with fractures classified as mild, moderate or severe.

**Results**: Greater symptoms of depression and greater catastrophic thinking were associated with a more limited association (moderation) between fracture severity and pain intensity and fracture severity and magnitude of activity intolerance, at lower levels of fracture severity 2 to 4 weeks after injury. Moderation 6 to 9 months after injury was observed only for pain intensity.

**Conclusions**: The observation that correspondence between pain intensity/activity intolerance and fracture severity is more limited when people have greater distress or misconceptions can help surgeons understand that relative divergence may signal mental health opportunities. Future quality improvement and research efforts can measure the benefit of strategies designed to anticipate and act on these opportunities.

### O10 Developing a multimedia patient-reported outcomes measure for low literacy patients

#### Chao Long^1,2,3^, Laura Beres^3^, Albert Wu^3^, Aviram Giladi^2^

##### ^1^Johns Hopkins Medicine, Baltimore, MD, USA. ^2^The Curtis National Hand Center, MedStar Union Memorial Hospital, Baltimore, MD, USA. ^3^Johns Hopkins Bloomberg School of Public Health, Baltimore, MD, USA

###### Correspondence: Chao Long

chaolong@jhu.edu

*Journal of Patient-Reported Outcomes* 5(1): O10.

**Objective:** Almost all patient-reported outcomes measures (PROMs) are text-based. This poses a barrier to accurate completion in low literacy populations. We developed the Multimedia Adaptation Protocol (MAP) for adapting validated, text-based PROMs to multimedia versions that can be self-administered in mixed literacy populations. We aimed to execute the first stage of the MAP, forward adaptation, to adapt the Patient Reported Outcomes Measurement Information System Upper Extremity Short Form (PROMIS-UE) to a multimedia version (mPROMIS-UE) for a mixed literacy hand surgery patient population in Baltimore, Maryland (USA).

**Methods:** Taking a community-engaged and human-centered design approach, forward adaptation included six phases undertaken in a serial, iterative fashion: planning with our community advisory board (CAB); direct observation; semi-structured interviews with patients, caregivers, and clinic staff; ideation; prototyping; and feedback. Direct observations were documented in memos which underwent rapid thematic analysis. Interviews were audio recorded and documented using analytic memos; framework analysis was used to identify inductive and deductive themes. Themes were further analyzed to identify personas and distilled into design challenges to guide ideation and prototyping that involved multidisciplinary research team members. To ensure the credibility of our findings we consulted our CAB and collected additional interviews including member-checking of initial findings post-analysis**.**

**Results:** We conducted 12 h of observations. We interviewed 17 adult English-speaking participants (12 patients, 3 caregivers, 2 staff) of mixed literacy (age 20–81 years). We identified two distinct user personas and three distinct literacy personas; we developed the mPROMIS-UE with these personas in mind. Themes from interviews were distilled into four broad design challenges surrounding literacy, customizability, convenience, and shame. We identified features (audio, animations, icons, avatars, progress indicator) that addressed the design challenges. These features were synthesized into a prototype that underwent four iterations of refinement.

**Conclusions:** We successfully adapted the PROMIS-UE to an mPROMIS-UE that addresses the challenges identified in a mixed literacy hand surgery patient population. This demonstrates the feasibility of adapting PROMs to multimedia versions. The mPROMIS-UE is ready to undergo the remaining stages of the MAP (back adaptation, qualitative evaluation, validation). A validated mPROMIS-UE will expand clinicians’ and investigators’ ability to capture patient-reported outcomes in mixed literacy populations.

### P11 Patient interpretations vary for PROMIS (Patient-Reported Outcomes Measurement Information System) upper extremity questions

#### Chao Long^1,2^, Laura Beres^3^, Albert Wu^3^, Aviram Giladi^1^

##### ^1^The Curtis National Hand Center, MedStar Union Memorial Hospital, Baltimore, MD, USA. ^2^Johns Hopkins Medicine, Baltimore, MD, USA. ^3^Johns Hopkins Bloomberg School of Public Health, Baltimore, MD, USA

###### Correspondence: Chao Long

chaolong@jhu.edu.

*Journal of Patient-Reported Outcomes* 5(1): P11.

**Objective:** The Patient-Reported Outcomes Measurement Information System Upper Extremity (PROMIS-UE) Short Form (SF) 7a includes seven items from the PROMIS-UE Item Bank v2.0, which includes items selected from the Physical Functioning Item Bank via consensus review and quantitative evaluation. We conducted a qualitative evaluation of the PROMIS-UE SF in patients with hand or upper extremity conditions. We sought to detect potential variability or challenges in comprehension, decision processes, response processes, and recall.

**Methods:** We conducted cognitive interviews with adult, English-speaking patients purposively sampled for mixed literacy from a hand and upper extremity clinic. Interviews were conducted in-person or by phone (based on patient preference during the COVID-19 era), audio-recorded, and documented in analytic memos. Interviews used a combination of “think aloud” and verbal probing techniques for each question (numbered PFA14r1, PFA34, PFA36, PFB13, PFB28r1, PFB34, PFM16). We utilized framework analysis to identify themes related to our goals. To ensure the credibility of our findings, we collected additional interviews that included member-checking of initial findings.

**Results:** Twelve patients 20–81 years old were interviewed. For PFA14r1 and PFB28r1 on lifting/carrying, patients were unsure how much “10 pounds/5 kg” weighed and tried to imagine household items of that weight. For PFA14r1, PFA34, PFB13, PFB28r1, and PFB34, patients were unsure whether to respond with their ability to perform the task with the injured extremity alone, with either the injured or healthy extremity, or with both. For PFA14r1, PFA34, and PFM16, patients indicated their response depends on specifics not mentioned in the question. For PFB28r1 and PFM16, patients struggled to recall performing these tasks with their injuries. Patients interpreted PFA36 as difficulty fitting splints into sleeves rather than functional limitations. Patients noted their responses vary depending on time of day. One patient noted uncertainty regarding whether she should answer questions based on learned adaptive techniques. The last five interviews included member-checking and confirmed the authenticity of our results.

**Conclusions:** Patients’ cognitive processes and interpretations varied for each question of the PROMIS-UE SF. This may undermine instrument reliability and validity. Two potential solutions include rewording questions or incorporating visuals to improve the instrument’s ability to communicate question intent.

### P12 Children's subjective health quality in ethnic minority areas in western China

#### Yipeng Lv^1^, Ye Gao^2^, Xu Liu^3^

##### ^1^Shanghai Jiaotong University, Shanghai, China. ^2^Maval Medical University, Shanghai, China. ^3^Naval Medical University, Shanghai, China

###### Correspondence: Yipeng Lv

epengl@163.com

*Journal of Patient-Reported Outcomes* 5(1): P12.

**Objective:** This research aims to explore the current status of children's physical health and subjective health at the underdeveloped ethnic minority areas in western China. Investigation on campus and family are also included in the research to figure out the main influencing factors and mechanism of subjective health in order to discover and provide early intervention for children's health risk factors.

**Methods:** A cross-sectional survey was conducted in August 2019 among 190 Tibetan primary school students in Maozhuang Township, Nangqian County, Qinghai Province. Structured forms were used to collect social and demographic information, family status, water accessibility, health habits, classmate relationship and homework burden. Patient-Reported Outcomes Measurement Information System (PROMIS) short forms, including Pediatric v1.1—Depressive Symptoms 8b, Pediatric v1.0—Anger 6a, Pediatric v1.1—Anxiety 8b, Pediatric v1.1—Anxiety 8b and Pediatric v1.0—Peer Relationships 8a were completed by each subjects. Raw score and T score of each short form were calculated in accordance with the score manual. Multivariate linear regression was performed to investigate potential factors associated with subjective health quality.

**Results:** The average values of the PROMIS five dimensions like Depression, anger, anxiety, fatigue and peer relationships of the research object are 58.9 ± 5.3, 53.3 ± 8.0, 58.1 ± 7.3, 52.8 ± 8.0 and 39.3 ± 6.6 separately. Compared with the norm standard value 50 ± 10, the p value is all lower than 0.0001, and the difference is statistically significant. Age, grade, family economic status, father's education level, classmate relationship and homework burden are statistically different in the subjective health of the research subjects.

**Conclusions:** Subjective health score of ethnic minority children in underdeveloped areas is lower than the norm score. Increasing grades will lead to a decline in children’s subjective health. Family environment and campus life will have an impact on children’s subjective health as well. During the campus education period, more attention is needed for caring students from poor economic condition family’s life, such as peer relationship, learning pressure, etc. Positive guidance should be given as soon as possible to help them build a better state of health.

### P13 Health-related outcomes differ among breast cancer patients with distinct patterns of symptom cluster

#### Qingmei Huang, Fulei Wu, Wen Zhang, Tingting Cai, Xian Zang

##### Fudan University, Shanghai, China

###### Correspondence: Qingmei Huang

hangqm@fudan.edu.cn

*Journal of Patient-Reported Outcomes* 5(1): P13.

**Objective:** Breast cancer patients who are undergoing chemotherapy often experience multiple, co-occurring symptoms such as pain, fatigue, anxiety, depression and sleep disturbance, which will deteriorate their health-related outcomes. This study was aimed to explore latent patterns of symptom cluster for breast cancer patients, and to compare the health-related outcomes differences among patients with different patterns.

**Methods:** Cross-sectional study design was used and pain, fatigue, anxiety, depression and sleep disturbance as symptom cluster were reported by participants using PROMIS measures. Latent Class Analysis (LCA) was used to explore the latent patterns of the above symptom cluster.

**Results:** 647 breast cancer patients who are under chemotherapy were finally included for analysis. Three latent profiles were identified by LPA, which were namely “high symptom cluster pattern” (24.2%), “high psychological symptom cluster pattern” (22.9%), and “low symptom cluster pattern” (52.8%), respectively. Compared to patients with “low symptom cluster pattern”, patients in the “high symptom cluster pattern” subgroup reported poorest health-related outcomes, including physical health, cognitive health and social health.

**Conclusions:** Breast cancer patients experienced different symptom cluster patterns, patients with high patterns should be given more attention to improve their outcomes.

## 14* Intentionally omitted

### O15 Associations between workers’ compensation status and preoperative PROMIS Global Health scores in orthopedic patients

#### Vinicius Antao^1^, Mark Fontana^1,2^, Miranda So^1^, Catherine MacLean^1^

##### ^1^Hospital for Special Surgery, Center for the Advancement of Value in Musculoskeletal Care, New York, NY, USA. ^2^Weill Cornell Medical College, Department of Healthcare Policy and Research, New York, NY, USA

###### Correspondence: Vinicius Antao

antaov@hss.edu

*Journal of Patient-Reported Outcomes* 5(1): O15.

**Objective:** To assess the association between workers’ compensation (WC) status and preoperative PROMIS Global Health scores among patients undergoing orthopedic surgery.

**Methods:** Between January 2017 and March 2021, we collected the PROMIS Scale v1.2 – Global Health (PROMIS-10) from patients undergoing orthopedic surgery in seven subspecialties. Patients received email notifications to complete the online survey up to 30 days before surgery, followed by a reminder 3 days before surgery. If the survey was not completed, nurses attempted to obtain responses through a phone call the night before surgery. We used linear regression to assess associations between PROMIS-10 T-scores (physical and mental health) and payer status (WC vs. non-WC) stratified by subspecialty, controlling for demographics, timing of PROM completion, and modality of collection.

**Results:** Among 121,426 total patients undergoing surgery, 95,887 completed the survey (78.9%), among whom 3,048 (3.2%) were on WC. Respondent subspecialties included Foot & Ankle (8.6%), Hand & Upper Extremity (8.1%), Joint Replacement (36.7%), Limb Lengthening (LL) (1.6%), Scoliosis (1.5%), Spine (11.5%), and Sports (31.8%). Average preoperative PROMIS-10 physical health T-scores were 45.6 among WC patients and 46.3 among non-WC patients (p < 0.001); mental health T-scores were 53.1 and 53.0, respectively (p = 0.510). After adjusting for covariates and stratifying by subspecialty, the largest average difference in T-scores between WC and non-WC patients was among those in Scoliosis (− 8.3 points), LL (− 5.9 points), and Spine (− 4.1 points). For mental health T-scores, the largest average difference was among those in LL (− 3.8 points), Spine (− 3.8 points), and Joint Replacement (− 2.2 points). Physical and mental health scores from WC and non-WC patients were not statistically significantly different among patients in Foot & Ankle (− 1.9; + 0.1), Hand (− 1.5; + 0.9), and Sports (− 0.4; + 0.9).

**Conclusions:** Compared with non-WC status, WC patients had significantly lower preoperative PROMIS-10 physical health and mental health T-scores in the Joint Replacement, Limb Lengthening, and Spine, and lower physical health scores in Scoliosis. In contrast, scores were not significantly different among patients in Foot & Ankle, Hand, and Sports.

### P16 Unpacking the Impact of chronic pain as measured by the Impact Stratification Score

#### Anthony Rodriguez^1^, Maria O. Edelen^2,1^, Patricia Herman^3^, Ron D. Hays^4^

##### ^1^RAND Corporation, Behavioral and Policy Sciences, Boston, MA, USA. ^2^Patient Reported Outcomes, Value and Experience (PROVE) Center, Department of Surgery, Brigham and Women’s Hospital, Boston, MA, USA. ^3^RAND Corporation, Behavioral and Policy Sciences, Santa Monica, CA, USA. ^4^Division of General Internal Medicine & Health Services Research, UCLA Department of Medicine, Los Angeles, CA, USA

###### *Correspondence: Anthony Rodriguez

anthonyr@rand.org

*Journal of Patient-Reported Outcomes* 5(1): P16.

**Objective:** Examine the dimensionality of the Impact Stratification Score (ISS) and support for its single total score, and evaluate the psychometric properties of ISS items.

**Methods:** The sample of 1677 chiropractic patients being treated for chronic lower back pain and chronic neck pain, had an average age of 49, 71% female, and 90% White. Study participants completed the PROMIS-29 v2.1 profile survey that contains the 9 ISS items. The ISS was evaluated using item-rest correlations, Cronbach’s alpha, factor analysis (i.e., correlated factors and bifactor models), and item response theory (IRT). Reliability indices and item properties were evaluated from bifactor and IRT models, respectively.

**Results:** Item-rest correlations were high (0.64–0.84) with a Cronbach’s alpha of 0.93. Eigenvalues suggested the possibility of two factors corresponding to physical function and pain interference/intensity. Bifactor model results indicated that data were essentially unidimensional, primarily reflecting one general construct (i.e., impact) and that after accounting for ‘impact’ very little reliable variance remained in the two group factors. General impact scores were reliable (omegaH = 0.73). IRT models showed that items were strong indicators of impact and provided information across a wide range of the impact continuum and offer the possibility of a shorter 8-item ISS. Finally, it appears that different aspects of pain interference occur prior to losses in physical function.

**Conclusions:** This study presents evidence that the ISS is sufficiently unidimensional, covers a range of chronic pain impact and is a reliable measure. Insights are obtained into the sequence of chronic pain impacts on patients’ lives.

## 17 * Intentionally omitted

### P18 Presenting PROMIS scores predict treatment selection for De Quervain’s Tenosynovitis

#### Gilbert Smolyak, Courtney Jones, Constantinos Ketonis

##### University of Rochester, Rochester, NY, USA

###### Correspondence: Gilbert Smolyak

gilbert_smolyak@urmc.rochester.edu

*Journal of Patient-Reported Outcomes* 5(1): P18.

**Objective:** Despite conservative management of de Quervain’s Tenosynovitis (DQT), many patients continue to suffer from recalcitrant symptoms necessitating surgical intervention. PROMIS scores at time of diagnosis might provide insights into success of non-operative management and predict necessity for surgical release.

**Methods:** Patients presenting to a tertiary academic medical center from 2014–2019 with a sole diagnosis of DQT were identified. Patients < 18 years old or that had other diagnosis were excluded. Patients were separated by treatment: physical therapy, injections, surgery or combinations thereof. Chi-square analysis was performed to identify confounding variables or demographic factors that affect treatment strategy. A multi-factor ANOVA analysis was performed to identify patterns in presenting PROMIS scores (PPS) and selection of initial treatment. Patient groups were then reorganized by the most invasive treatment pursued, the analysis was repeated, and t-test analysis confirmed statistical differences. Patients without a PPS were excluded from statistical tests involving PROMIS.

**Results:** Of the 1529 patients who met inclusion/exclusion criteria, 729 of which had PPS. For initial treatment, 119 (7.8%) patients chose PT, 831 (54.3%) chose an injection, 129 (8.4%) chose surgery, and 450 (29.4%) had no intervention. Of the patients who received treatment 85 (7.9%) had only PT, 695 (64.4%) received at least one injection during treatment, and 299 (27.7%) eventually had surgery. Significant differences in PPS between patients of initial treatment group were not of clinically important difference. However, patients that eventually required surgery had significantly lower PF (p = 3.751e-08), higher PI (p = 1.431e-08) and higher PD (p = 0.0146) when compared to those that only had injections.

**Conclusions:** PROMIS survey results could be used to identify patients that are likely to fail non-operative intervention for DQT. Survey response rates were much higher from patients choosing more invasive interventions and older patients tended to choose more invasive treatments as their initial management. While there were no clinically significant differences in PPS between patients choosing PT, injection, or surgery as their initial management, patients that eventually necessitated surgery had significantly lower PF, higher PI, and higher PD PPS than those who chose injection.

### P19 Reliability, validity of PROMIS Social Isolation short form in Chinese family caregivers of cancer patients

#### Fulei Wu^1^, Qingmei Huang^1^, Yang Yang^2^, Li Ning^3^, Limin Zhu^1^, Tianai Fan^1^, Changrong Yuan^1^

##### ^1^Fudan University, School of Nursing, Shanghai, China. ^2^Oncology Hospital of Fudan University, Shanghai, China. ^3^Hangzhou First People's Hospital, Hangzhou, China

###### Correspondence: Fulei Wu

wufulei@fudan.edu.cn

*Journal of Patient-Reported Outcomes* 5(1): P19.

**Objective:** Being diagnosed of cancer is a huge challenge not only for patients themselves, but also their families, especially the main caregivers. Because of heavy care as well as financial burden, the life trajectory of the caregivers is even transformed into a ‘patient-centered’ circle, which limits their original social roles and social activities, thus increase their sense of social isolation and influencing their quality of life. It is of great necessary to assess social isolation of caregivers of cancer patients. However, limited scale has been designed for this group of population. Patient-reported Outcomes Measurement Information System (PROMIS) social isolation short form (SI-SF) is one of widely used scales measuring social health across diseases, and may be a potential measurement tool to assess social isolation of family caregivers of cancer patients.

**Methods:** By convenience sampling, a total sample of 110 family caregivers of cancer patients were recruited from 2 hospitals in Shanghai and Hangzhou. A Self-designed social demographic and disease information questionnaire and PROMIS SI-SF were used for data collection. Item-total correlation, internal consistency, and split-half reliability was used to examine the reliability, confirmatory factor analysis (CFA) was used to examine construct validity. Statistical software used were SPSS 21.0 and Mplus 7.0. P < 0.05 was considered statistically significant.

**Results:** The item-total correlation coefficient (r) ranged from 0.447 to 0.883 (All P < 0.01). The Cronbach's α coefficient was 0.958, and the Gunttman split-half coefficient was 0.915, indicating that PROMIS SI-SF had high internal consistency and split-half reliability. The result of CFA showed that PROMIS SI-SF was unidimensional with acceptable but not ideal model fit index. Chi-Square/df was (141.121/75) < 2, comparative fit index (CFI) and Tucker-Lewis index (TLI) were 0.922 and 0.906, respectively (all > 0.9), Standardized Root Mean Square Residual (SRMR) value was 0.051, Root Mean Square Error Of Approximation (RMSEA) was 0.09.

**Conclusions:** PROMIS SI-SF is reliable and valid for assessing social isolation in Chinese family caregivers of cancer patients. Larger sample is needed to further examine the stableness of the unidimensional structure of the short form.

### P20 Feasibility, measurement properties and relevance of PROMIS item banks in adults with haemophilia – preliminary results

#### Isolde A.R. Kuijlaars^1^, Lorynn Teela^2^, Lize F.D. van Vulpen^1^, Merel A. Timmer^1^, Michiel Coppens^3^, Samantha C. Gouw^4,5^, Marjolein Peters^4^, Marieke J.H.A. Kruip^6^, Marjon H. Cnossen^7^, Jelmer Muis^4,2^, E. Shannon van Hoorn^8^, Lotte Haverman^2^, Kathelijn Fischer^1^

##### ^1^Van Creveldkliniek, University Medical Center Utrecht, Utrecht University, Utrecht, Netherlands. ^2^Amsterdam University Medical Centers, University of Amsterdam, Emma Children’s Hospital, Child and Adolescent Psychiatry & Psychosocial Care, Amsterdam Reproduction and Development, Amsterdam Public Health, Amsterdam, Netherlands. ^3^Department of Vascular Medicine, Amsterdam Cardiovascular Sciences, Amsterdam University Medical Centers, University of Amsterdam, Amsterdam, Netherlands. ^4^Emma Children’s Hospital, Amsterdam University Medical Centers, University of Amsterdam, Pediatric Hematology, Amsterdam, Netherlands. ^5^Department of Clinical Epidemiology, Leiden University Medical Center, Leiden, Netherlands. ^6^Erasmus MC, University Medical Center Rotterdam, Department of Hematology, Rotterdam, Netherlands. ^7^Erasmus MC, University Medical Center Rotterdam, Department of Pediatric Oncology and Hematology, Rotterdam, Netherlands. ^8^Erasmus MC, University Medical Center Rotterdam, Department of Public Health, Rotterdam, Netherlands.

###### Correspondence: Lotte Haverman

l.haverman@amsterdamumc.nl

*Journal of Patient-Reported Outcomes* 5(1): P20.

**Objective:** Legacy haemophilia outcome measures may be too long and sometimes have floor- or ceiling effects and irrelevant questions. Patient Reported Outcomes Measurement Information System (PROMIS) item banks use Computer Adaptive Tests (CAT) to enable more efficient outcome assessment than legacy instruments. The aim of this study was to evaluate the feasibility, measurement properties and relevance of nine PROMIS CATs and short forms (SFs) in persons with haemophilia (PWH).

**Methods:** Dutch adult PWH completed nine PROMIS item banks electronically as CATs or SFs: ‘physical function’, ‘pain interference’, ‘depression’, ‘anxiety’, ‘ability to participate in social roles and activities’, ‘satisfaction with social roles and activities’, ‘fatigue’, ‘self-efficacy for managing medications and treatment’ and ‘self-efficacy for managing symptoms’. Feasibility was assessed by number of items answered per CAT and floor-/ceiling effects for all measures. Construct validity was studied by testing hypotheses about the relationship of PROMIS items banks with the legacy instruments Haemophilia Activities List, RAND-36, HEP-test-Q, veritas-PRO and PAM-13 (convergent validity) and expected differences between subgroups (known-group validity). The reliability of the CATs was evaluated by calculating the proportion of T-scores with an SE ≤ 3.2. Relevance of item banks was determined by proportions of limited scores.

**Results:** Overall, 142/373 of invited PWH (mean age 47 years [range 18–79], 49% severe haemophilia, 46% received prophylaxis) responded. For the CATs, mean number of items answered per item bank varied from 5 (range 3–12) to 9 (range 5–12), with floor effects in ‘pain interference’ (26% lowest scores) and ‘depression’ (18% lowest scores). Construct validity and reliability in PWH were good for ‘physical function’, ‘pain interference’, ‘satisfaction with social roles and activities’ and ‘fatigue’. Limited scores were most prevalent in the CATs ‘pain interference’ (33%) and ‘physical function’ (38%). The self-efficacy SFs with 8 items showed ceiling effects (22–28% maximum scores) and showed no relation with the legacy instruments.

**Conclusions:** The PROMIS CATs ‘physical function’, ‘pain interference’ and ‘satisfaction with social roles and activities’ and ‘fatigue’ are feasible and valid tools in PWH and preferred to the legacy instruments based on fewer items and less floor- and ceiling effects.

### P21 PROMIS provides a broader overview of HRQL than the ESSPRI in Sjogren’s Syndrome

#### Dana DiRenzo, Susan Robinson, Clifton O. Bingham, III, Alan N. Baer, Thomas Grader-Beck

##### Johns Hopkins University, Baltimore, MD, USA

###### Correspondence: Thomas Grader-Beck

tgrader1@jhmi.edu

*Journal of Patient-Reported Outcomes* 5(1): P21.

**Background**: Sjogren’s Syndrome (SS) is an autoimmune disease affecting the exocrine glands that has considerable impact on health-related quality of life (HRQL). The Patient Reported Outcome Measurement Information System (PROMIS) provides universal HRQL instruments, but has not been previously implemented in SS.

**Methods:** A cross-sectional evaluation was performed on completed questionnaires of consecutive adult patients during visits to a multidisciplinary Sjogren’s clinic between March 2018-February 2020. Questionnaires included PROMIS short-forms (depression 4a, anxiety 4a, fatigue 8a, physical function 4a, pain interference 8a (PI), sleep disturbance 4a (sleep), participation in social roles and activities 8a) and the European League Against Rheumatism (EULAR) Sjogren’s Syndrome Patient Reported Index (ESSPRI). Patients were either classified as SS by 2016 ACR/EULAR criteria or otherwise labeled as sicca not otherwise specified (NOS) and used as a comparison group. Descriptive statistics were calculated for disease-related and sociodemographic variables and Pearson correlation was used to evaluate the relationship between subdomains of the ESSPRI and PROMIS. Uni- and multivariable linear regression (MVR) models were used to evaluate predictors of PROMIS fatigue, PI, and social participation.

**Results:** 227 SS patients and 85 patients with sicca NOS were included. Mean (SD) PROMIS T-scores for PI (56.9 (11.0)), fatigue (57.2 (10.6)), and physical function (44.2 (9.8)) in SS patients were at least ½ SD worse than US population normative values. Among SS patients PROMIS PI (r = 0.72) and fatigue (r = 0.80) highly correlated with respective ESSPRI pain and fatigue sub-domains. Fatigue (β = − 0.610, p < 0.001) and PI (β = − 0.185, p < 0.001), but not dryness or mood disturbance, were the strongest predictors of social participation in MVR in this SS cohort.

**Conclusions:** In our SS cohort, PROMIS PI and fatigue scores correlated highly with respective ESSPRI domains. Fatigue but not dryness was found to be the strongest predictor of social participation. Given the ability of PROMIS instruments to evaluate physical, mental, and social function that would otherwise not be ascertained through the ESSPRI, these questionnaires should be considered as supplement in evaluation of SS.

### P22 The use of pediatric PROMIS item banks in Dutch boys with haemophilia

#### Lotte Haverman

##### Amsterdam MC, Amsterdam, Netherlands

###### Correspondence: Lotte Haverman

l.haverman@amsterdamumc.nl

*Journal of Patient-Reported Outcomes* 5(1): P22.

**Objective**: Frequently used disease specific Patient Reported Outcome Measures (PROMs) in pediatric haemophilia are experienced as a burden due to their length and sometimes irrelevant questions. Patient Reported Outcomes Measurement Information System (PROMIS) item banks using short forms (SF) or Computerized Adaptive Testing (CAT) could solve this problem. The objective of this study is to assess the psychometric properties and feasibility of eight PROMIS item banks within a clinical sample of boys with haemophilia.

**Methods:** In this multicenter study, all boys with haemophilia (mild, moderate, severe hemophilia A/B, aged 8–17 years) from six Dutch Haemophilia Treatment Centers will be invited to participate. For assessment of convergent validity the PROMIS item bank T-scores will be compared to subscales of the Haemophilia Quality of Life Questionnaire for Children (HaemoQoL) and to the Pediatric Haemophilia Activities List (PedHAL) by using Pearson’s *r* with Normative data, at which *r* ≥ 0.70 is considered acceptable (Table 1). To ensure a power of > 0.8, a sample of n ≥ 64 is needed. Reliability of the PROMIS item banks was expressed as standard error of theta (SE(θ)), at which an SE(θ) < 0.32 corresponds to a reliability of 0.90. The proportion of reliable (SE(θ) < 0.32) T-scores within each item bank will be reported. Regarding feasibility, the number of completed items will be reported.

**Results:** Regarding convergent validity, *r* is hypothesized between 0.5—0.9 for all correlations between the domains mentioned in Table 1. The proportion reliable T-scores is expected to be good for all PROMIS item banks, based on Dutch studies in children from the general population and a clinical sample (Juvenile Idiopathic Arthritis). We expect the PROMIS item banks to be more feasible in terms of number of items completed.

**Conclusions:** When the pediatric PROMIS item banks display good convergent validity with disease-specific legacy instruments, good internal consistency and feasibility in a clinical sample of Dutch boys with haemophilia, PROMIS can be used in research and clinical care with lower questionnaire-related burden.

### O23 Are you better yet? Using plausible values to determine true change with PROMIS measures

#### Felix Fischer^1^, Jay Verkuilen^2^, Emily Ho^3^

##### ^1^Charité Universitätsmedizin Berlin, Berlin, Germany. ^2^CUNY graduate center, New York, NY, USA. ^3^Northwestern University, Chicago, IL, USA

###### Correspondence: Felix Fischer

felix.fischer@charite.de

*Journal of Patient-Reported Outcomes* 5(1): O23.

**Objective:** One core advantage of PROMIS measures is that each estimate of the latent trait is associated with a standard error, reflecting uncertainty in the measurement. Such uncertainty needs to be acknowledged and quantified, in particular when assessing individual patients over time. In this study, we use plausible values to analyze true scores rather than observed scores. We then analyze the probability of true within-individual change and illustrate the use of plausible values in the analysis of real-world PRO data.

**Methods:** We used a freely available dataset of stable and exacerbated COPD patients (N = 185), [1] which provided individual’s physical function and fatigue PROMIS T-scores over a course of 21 weeks. At each measurement, we imputed 1000 plausible values from a normal approximation to the PROMIS T-scores’ posterior distribution. Plausible values were then used to calculate probability of true change from baseline and the previous assessment, on individual and sample level. We also compared 4-item, 8/10-item short forms and computer-adaptive test in their performance to determine true change with 80%, 90% and 95% certainty across the T-Score metric.

**Results:** We observed that at the end of the study, in the exacerbated group, 47.5% of participants achieved a certain (T-Score Difference t1-t2 < 0, p > 95%) improvement in fatigue from baseline compared to 26.5% in the stable group. Comparison of short forms and CATs of physical function and fatigue suggests that CATs have the most favourable properties, with a constant theta change of approximately 5 points reflecting a 95% probability of true improvement. For short forms, theta change associated with 95% certainty of true change can vary considerably depending on the T-score.

**Conclusions:** Plausible values offer a flexible way to include measurement error in analysis of individuals and on a group level, and offer a useful complement to existing distribution-based approaches by providing an assessment of probability of true change. This method facilitates ease of interpretation, and allows for a finer-grained comparison of improvement or decline than analysis of observed scores.

References

[1] Yount, S.E., Atwood, C., Donohue, J., Hays, R.D., Irwin, D., Leidy, N.K., Liu, H., Spritzer, K.L. and DeWalt, D.A. (2019). Responsiveness of PROMIS to change in chronic obstructive pulmonary disease. *Journal of Patient-Reported Outcomes*, 3(1).

### P24 Differential item functioning of PROMIS physical functioning ceiling items across Argentina, Germany, and the U.S.

#### Constantin Yves Plessen^1,2^, Claudia Hartmann^1^, Marilyn Heng^3^, Rodrigo Pesantez^4^, Felix Fischer^1^, Matthias Rose^1^

##### ^1^Department of Psychosomatic Medicine, Center of Internal Medicine and Dermatology, Charité—Universitätsmedizin Berlin, Berlin, Germany. ^2^Department of Clinical, Neuro-, and Developmental Psychology, Vrije Universiteit Amsterdam, Amsterdam, Netherlands. ^3^Department of Orthopaedic Surgery, Orthopaedic Trauma Service, Massachusetts General Hospital, Boston, MA, USA. ^4^Department of Orthopedic Surgery, Fundación Santa Fe de Bogota, Universidad de los Andes, Bogota, Colombia

###### Correspondence: Constantin Yves Plessen

constantin-yves.plessen@charite.de.

*Journal of Patient-Reported Outcomes* 5(1): P24.

**Objective:** Thirty-five new items were introduced to the Patient-Reported Outcomes Measurement Information System (PROMIS) Physical Functioning item bank to extend the range of measurement. Differential item functioning (DIF) can introduce biases to inter-country comparisons, potentially leading to systematically different physical function scores. Individuals with the same ‘true’ underlying physical ability would score systematically different due to specific cultural contexts or language differences. Therefore, we investigated these items in three general population samples and assessed the validity of their German and Spanish translations.

**Methods:** We collected P.F. data from 3601 persons from the general population in the USA, Argentina, and Germany. DIF was assessed with logistic ordinal regression models, and Nagelkerkes’ pseudo *R*^2^-change of > 0.02 was chosen as the critical cutoff value indicating DIF. The impact of DIF on item scores and the *T*-scores were examined by inspecting both the item characteristic curves (ICCs) and test characteristic curves (TCCs).

**Results:** We included 1001 participants from Argentina (*M*_*theta*_ = 0.23, *SD*_*theta*_ = 0.79; *M*_*age*_ = 35.6; 51% female), 1000 from Germany (*M*_*theta*_ = 0.11, *SD*_*theta*_ = 1.02; *M*_*age*_ = 44.9; 52% female), and 1600 from the U.S. (*M*_*theta*_ = 0.00, *SD*_*theta*_ = 1.22; *M*_*age*_ = 44.3; 58% female). 2 (Germany vs. the U.S.) respectively 4 (Argentina vs. the U.S.) out of 35 items were flagged for DIF. Most of the items that showed DIF had *R*^2^ values that only marginally exceeded the critical value of 0.02. All these items showed uniform DIF. The TCCs suggested that the magnitude and impact of DIF on the test-scores was negligible for all items. After correcting for potential DIF, both Germany (*M*_*theta*_ difference = 0.098, *t*(2393.4) = 2.57, *p* = 0.0103) and Argentina (*M*_*theta*_ difference = 0.281,* t*(2598.2) = 7.91, *p* < 0.001) had slightly higher scores than the U.S.

**Conclusions:** Our study adds to the evidence that PROMIS physical functioning items are universally applicable across general populations from Argentina, Germany, and the U.S.

### P25 Targeting conceptual equivalence in the Norwegian linguistic validation of Pediatric and Parent Proxy PROMIS items

#### Emily Parks-Vernizzi^1^, Abigail Boucher^1^, Benjamin Arnold^1^, Helena Correia^2^, Ida Sletten^3^, Andrew Garratt^4^

##### ^1^FACITtrans, Ponte Vedra, FL, USA. ^2^Northwestern University, Feinberg School of Medicine, Department of Medical Social Sciences, Chicago, IL, USA. ^3^Oslo University Hospital, Division of Orthopaedic Surgery, Oslo, Norway. ^4^Norwegian Institute of Public Health, Oslo, Norway

###### Correspondence: Emily Parks-Vernizzi

eparks@facit.org

*Journal of Patient-Reported Outcomes* 5(1): P25.

**Objective:** The purpose of this study was to translate and linguistically validate four PROMIS Pediatric item banks (Anxiety, Depressive Symptoms, Peer Relationships, Upper Extremity Function), the Pediatric Proflie-25, Pediatric Global Health Scale 7 + 2 and Parent Proxy counterparts into Norwegian highlighting linguistic issues encountered during the process.

**Methods:** We translated 108 PROMIS Pediatric items and 109 Parent Proxy items using the FACIT methodology – a standardized iterative process of forward- and back-translation, expert review, harmonization, and cognitive interviewing. The translation team were native Norwegian-speakers from Norway. 15 Norwegian-speaking parent–child dyads from the general population assessed the relevance, understandability, and appropriateness of the translations. A pragmatic qualitative analysis of cognitive interviews determined the linguistic equivalence of each translation and provided insight into the relevance of the concepts for each population.

**Results:** The study sample consisted of 15 native Norwegian-speaking children (7 girls, 8 boys) with a mean age of 13 (8–17) and 15 native Norwegian-speaking adults (11 women, 4 men) with a mean age of 42 (33–47) in Oslo, Norway. Revisions to particular concepts were made to Pediatric items where respondent commentary revealed misunderstandings (Pediatric Profile-25: “pay attention”, “one block”, Pediatric Global Health Scale: “rate”, “mood”) and to corresponding Parent Proxy items to maintain consistency. One additional revision was required to the Parent Proxy Global Health: “feel sad”. Upon completion of the cognitive interview analysis, translations were reviewed by the Norwegian PROMIS National Center and collaborators in Oslo to further refine items’ verbiage.

**Conclusions:** The Norwegian language PROMIS Pediatric and Parent Proxy items are conceptually equivalent to the English source. Concurrent assessment of children’s and parents’ item interpretation confirmed consistent understanding between pediatric and proxy populations. Inclusion of PROMIS National Centers in the translation process was instrumental in fine tuning particular nuances for both populations and is recommended in future translation work. These Norwegian Pediatric and Parent Proxy items are acceptable for use in international research, clinical trials and practice.

### P26 Translation and linguistic validation of Dutch-Flemish PROMIS Item Banks: Cognitive Function, Itch—Interference, and Self-Efficacy

#### Emily Parks-Vernizzi^1^, Abigail Boucher^1^, Benjamin Arnold^1^, Valentijn Zonjee^2^, Helena Correia^3^, Isolde Kuijlaars^4^, Kathelijn Fischer^4^, Lotte Haverman^5,6^, Caroline Terwee^5^

##### ^1^FACITtrans, Ponte Vedra, FL, USA. ^2^OLVG, Amsterdam, Netherlands. ^3^Northwestern University, Feinberg School of Medicine, Department of Medical Social Sciences, Chicago, IL, USA. ^4^Van Creveldkliniek, University Medical Center Utrecht, Utrecht University, Utrecht, Netherlands. ^5^Amsterdam UMC, Amsterdam, Netherlands. ^6^Emma Children’s Hospital, Amsterdam, Netherlands

###### Correspondence: Emily Parks-Vernizzi

eparks@facit.org

*Journal of Patient-Reported Outcomes* 5(1): P26.

**Objective:** The purpose of this study was to translate and linguistically validate five adult PROMIS item banks (Cognitive Function, Cognitive Function – Abilities, Itch – Interference, and two Self-Efficacy for Managing Chronic Conditions item banks: Managing Medications/Treatment and Managing Symptoms) in Dutch-Flemish and report on challenges and solutions encountered during the process.

**Methods:** We translated 115 adult PROMIS items using the FACIT methodology, which is a standardized iterative process of forward- and back-translation, expert review, harmonization, and cognitive interviewing. The translation team consisted of native Dutch-speaking linguists from Belgium and the Netherlands. As an additional quality measure, prior to cognitive interviews the Dutch-Flemish PROMIS National Center (PNC) reviewed all translations to confirm fluency, harmonization with previous translations, and offer suggestions relating to the items’ usage in clinical settings. Eighteen Dutch-speaking participants from the general population evaluated the relevance, comprehensibility, and appropriateness of the items. We conducted qualitative analysis of cognitive interviews to evaluate the linguistic equivalence of each translated item and provide insight into the relevance of the concepts.

**Results:** The sample consisted of 18 native Dutch-speaking adults (8 women, 10 men) from Belgium and the Netherlands with a mean age of 49 (18–75) years. During the translation phase, the concepts “I was tired of people asking” (Itch – Interference), “I can manage,” and “manage my symptoms” (Self-Efficacy item banks: Managing Medications/Treatment and Managing Symptoms) required adjustments to convey the intended meaning more accurately and to harmonize with existing translations. Cognitive interviews revealed that of the 115 items translated, only one required revision (Cognitive Function: “My thinking has been foggy”). The remaining 114 items required no revisions, and all items were found to be relevant.

**Conclusions:** The Dutch-Flemish PROMIS item banks are considered conceptually equivalent to the English. Short forms are ready for use in international research, clinical trials, and practice. Full banks will be validated with Dutch/Flemish patients before implementation as CAT. Inclusion of the Dutch-Flemish PNC is recommended for harmonization with existing translations and to maintain the link between linguistic choices and applied usage in clinical settings.

### P27 Impact of COVID-19 Infection on health-related quality of life: differences between Latinos and non-Latinos

#### Case Kathleen^1,2^, Sarah Lill^1^, Alexandra Howell^3^, James Bridges^1,2^, Daniel MacCarthy^4^, Paula Winkler^1,5^, Joel Tsevat^1,2^

##### ^1^Center for Research to Advance Community Health (ReACH), Joe R. and Teresa Lozano Long School of Medicine, The University of Texas Health Science Center at San Antonio, San Antonio, TX, USA. ^2^Department of Medicine, Joe R. and Teresa Lozano Long School of Medicine, The University of Texas Health Science Center at San Antonio, San Antonio, TX, USA. ^3^3Joe R. and Teresa Lozano Long School of Medicine, The University of Texas Health Science Center at San Antonio, San Antonio, TX, USA. ^4^Department of Population Health Sciences, Joe R. and Teresa Lozano Long School of Medicine, The University of Texas Health Science Center at San Antonio, San Antonio, TX, USA. ^5^South Central Area Health Education Center, School of Nursing, The University of Texas Health Science Center at San Antonio, San Antonio, TX, USA

###### Correspondence: Case Kathleen

casek1@uthscsa.edu

*Journal of Patient-Reported Outcomes* 5(1): P27.

**Objective:** We sought to investigate the health, socioeconomic, and behavioral impacts of COVID-19 among an ethnically diverse population of COVID-19 survivors in Texas, and to compare effects in Latinos versus non-Latinos.

**Methods:** In December 2020, we surveyed (in English or Spanish) patients who had had COVID-19 infection 3–9 months earlier. Measures included 1) the PROMIS-29 + 2 health profile, 2) the CAIR Pandemic Impact Questionnaire (C-PIQ), and 3) items addressing social determinants of health. Bivariate analyses included chi-square tests, Wilcoxon rank-sum tests, and T-tests; generalized linear models were conducted for multivariable analyses.

**Results:** Survivors of COVID-19 (n = 145; [mean {range} age 45 {18–85} years, 70% female, 76% Latino) reported compromised health-related quality of life, scoring worse than general population norms on all PROMIS-29 + 2 domains and having a mean [SD] health utility = 0.33 [0.24]). Latinos reported significantly lower health utility (0.30[0.22] versus 0.45[0.28] p = 0.005) and worse outcomes on 4 of the 8 PROMIS domains as compared with non-Latinos: Anxiety (60.4[10.5] versus 52.1[11.0], p = 0.01), Depression (55.3[10.6] versus 49.9[10.2], p = 0.02), Sleep Disturbance (56.5[9.0] versus 51.0[10.6], p = 0.01) and Cognitive Function – Abilities (m = 46.9[8.6] versus 51.6[8.4], p = 0.01). Latinos more commonly reported impacts of COVID-19 on social determinants of health such as finances (53% versus 21%, p = 0.003) and conflict within the home or family (18% versus 7%, p = 0.01). Multivariable regression analyses suggested that ethnic disparities in Depression, Anxiety, Seep Disturbance, and Cognitive Function were partially attributed to financial concerns. Conversely, Latinos had significantly higher C-PIC scores (CAIR Growth Score = 8.2[4.7] versus 5.3[4.2], p = 0.003), with higher scores on items such as “COVID-19 strengthened your relationships,” “increased appreciation of life,” and “created spiritual change.”

**Conclusions:** COVID-19 has detrimental but differential impacts on patient-reported outcomes and social determinants of health among Latinos compared with non-Latinos, but Latinos may experience more post-traumatic growth. These findings highlight the ongoing need to address health disparities in not only infection, but also recovery from COVID-19. Furthermore, as Latinos also reported some more positive impacts due to the COVID-19 pandemic, future research should examine whether personality characteristics (e.g. resilience) mediate the impact of COVID-19 on health-related quality of life.

### P28 PROMIS validation in Myasthenia Gravis population

#### Kevin Rivera^1^, David Lacomis^2^, Parthasarathy Thirumala^2^, Janel Hanmer^2^

##### ^1^University of Pittsburgh School of Medicine, Pittsburgh, PA, USA. ^2^UPMC, Pittsburgh, PA, USA

###### Correspondence: Kevin Rivera

kriveraa1995@gmail.com

*Journal of Patient-Reported Outcomes* 5(1): P28.

**Objective:** Myasthenia Gravis (MG) characterized by generalized weakness commonly due to autoantibodies blocking acetylcholine receptors (AchR) resulting in symptoms like ptosis, diplopia, dysphagia, and dysarthria. The MG-QoL, specifically designed for MG, has historically been used to measure this population’s QoL. To our knowledge, PROMIS has not been evaluated for use in patients with MG. In our evaluation of PROMIS in this population we expect PROMIS anxiety, depression, fatigue, social roles, physical function, and cognitive function scores will be strongly correlated with MG-QoL scores in patients with MG. Also, in clinical subgroups with significant differences in MG-QoL scores, strongly correlated PROMIS scores will be expected to show significant differences.

**Methods:** Starting June 2018, 8 PROMIS domains have been collected as part of routine clinical practice in our neurology clinics. Measures are automatically assigned by the electronic health record and are usually completed by tablet while in waiting rooms. Subjects completed measures before March 2020 and have an MG billing code. Other data elements were extracted from the e-record (i.e., demographics) or by abstraction (i.e., comorbid conditions). Pearson correlations were calculated between scores. Differences in scores between clinical subgroups was evaluated using linear regression. Correlations are interpreted using Cohen’s effect sizes and coefficients are considered statistically significant if p < 0.05.

**Results:** Data collection is complete for 200/360 patients (131/200 have multiple visits) from 12 clinical sites and 37 providers. The average age is 65 and 54% are female. The MGQoL and PROMIS scores had medium strength correlations for cognitive function, sleep disturbance, and depression, and strong correlations for anxiety, fatigue, pain interference, social roles, and physical function. The strongest correlations are social roles and fatigue. All correlations had a p < 0.0001. In relevant clinical subgroups, whenever MGQoL was statically significant PROMIS physical function and social roles were also statistically significant, except for the pyridostigmine treatment category. Conversely, several subgroups had statistically significant PROMIS scores but not MGQoL scores.

**Conclusions:** PROMIS and MGQoL measure many of the same constructs, with strong corrleations between MGQoL and many PROMIS domains. PROMIS shows more differences in QoL in clinically important subgroups of interest than MGQoL in patients with MG.

### P29 Are pediatric PROMIS general health scales ready for use in routine care? A systematic review

#### Emma Stallwood^1^, Andrea Monsour^1^, Ami Baba^1^, Nancy Butcher^1,2^, Martin Offringa^1,2^

##### ^1^The Hospital for Sick Children, Toronto, Canada. ^2^University of Toronto, Toronto, Canada

###### Correspondence: Emma Stallwood

estallwood6@gmail.com

*Journal of Patient-Reported Outcomes* 5(1): P29.

**Objective:** To determine if three pediatric *Patient-Reported Outcomes Measurement Information System* (PROMIS) questionnaires have sufficient measurement property evidence to be recommended for use in routine outcome monitoring as part of clinical care.

**Methods:** We assessed the (1) *PROMIS Parent Proxy short form v1.0- cognitive function 7a* questionnaire for ages 8 to 17 years; (2) *PROMIS Parent Proxy Scale v1.0-Global health 7* + *2* questionnaire for ages 5 to 17 years; and (3) *PROMIS Pediatric Scale – Global health 7* for ages 8 to 17 years using the COnsensus-based Standards for the selection of health Measurement INstruments (COSMIN) guidelines to (i) identify studies that evaluated the measurement properties of these questionnaires, (ii) evaluate the methodological quality of the included studies using the COSMIN Risk of Bias checklist, (iii) determine the sufficiency of each measurement property using COSMIN criteria for Good Measurement Properties, and (iv) assess the overall quality of evidence for each measurement property using modified GRADE criteria to determine if these outcome measurement instruments (OMIs) have sufficient evidence to be recommended for use. We searched the HealthMeasures website, MEDLINE, Embase, PsycINFO, and Web of Science and Google Scholar to identify eligible studies that assessed any of nine different aspects of reliability, validity, and responsiveness in participants < 18 years or caregivers of this age, as appropriate for each questionnaire.

**Results:** Across the 6 measurement property studies we included in this review, there were 4818 children and 5459 parents. The three PROMIS OMIs had “high quality of evidence” for sufficient structural validity and internal consistency but “low quality of evidence” for sufficient content validity, meeting COSMIN’s minimum standard for recommending their use. The quality of evidence was downgraded due risk of bias from the reporting of methods in the content validity studies. These findings apply to children ages 8 to 17 years, except the PROMIS Parent Proxy Scale v1.0 – Global Health 7 + 2 which is recommended for ages 5 to 17 years.

**Conclusions:** The PROMIS OMIs assessed in this review measure their intended constructs but only for their intended age group. We recommend that future research follows the COSMIN measurement properties reporting guide to avoid reporting biases.

### P30 Effects of age and cognitive impairment on PROMIS-57 Profile responses

#### George Jay Unick, Veronica Fallon, Kimberly Bamberger, Philip Heiser, Julia Biernot, Ann Gruber-Baldini, Lisa Shulman

##### University of Maryland, Baltimore, MA, USA

###### Correspondence: George Jay Unick

junick@gmail.com

*Journal of Patient-Reported Outcomes* 5(1): P30.

**Objective** The validity of PROMIS measures in older adults, especially age 80 + with and without cognitive impairment has not been rigorously studied. Our ongoing NIH study, PROMIS Profile Measures in Older Adults examines the effects of age and cognitive impairment on the validity of PROMIS-57 Profile responses using: 1) cognitive interviews and 2) quantitative testing of cognitive and age-related item bias.

**Methods** We conducted cognitive interviews in 38 older adults with MoCA 10–30 to explore responses on the PROMIS-57. Each interview was coded by two coders with conflicts resolved by consensus. Codes were grouped into thematic categories, including cognitive/age-related difficulty, problems with interpreting questions, and item-specific problems. In this abstract we describe items with problems related to cognition.

**Results** The sample was 47% female, 83% White/14% Black, 32% MoCA 10–17, and 47% age 80 + . Categories of cognitive problems included difficulty recognizing disability and difficulty associating words with personal experience. One example of difficulty recognizing disability was: Question- *Are you able to go up and down stairs at a normal pace?* An 80-year-old (MoCA 26) responded “without any difficulty”, later describing, *“I need to make sure I have four appendages touching things at all times. So, climbing I use two hands, two feet, and a cane*.” One example of difficulty associating words with experience was: Question- *I have trouble doing all of my usual work (including work at home).* Unemployed participants had difficulty reframing “work” as their daily activities. When asked what “usual work” meant, a 70-year-old (MoCA 14) said they didn’t know. When asked the follow-up question, *What are usual things you do every day?*, the response was, “*Get dressed”*. An 81-year-old (MoCA 11), disagreed that scrubbing floors and moving heavy furniture were examples of heavy work. Yet when asked, *Can you think of better examples of heavy work?,* they responded, “*walking”*.

**Conclusions** Cognitive impairment and age-related role change effect patient responses due to problems with interpretation of words, associating words with experience and perception of disability. Our presentation will highlight PROMIS-57 items that were more or less robust to cognitive- and age-related impairment.

### O31 PROMIS translation platform – web application for supporting and supervising the translation methodology regime

#### Piotr Bosak1, Ida Jokisz1, Maciej Malewicz1, Jakub Michalski1, Agnieszka Żukowska2, Wojciech Glinkowski2,3,4,5

##### ^1^Polish Japanese Academy of Information Technology, Warsaw, Poland. ^2^Polish Telemedicine and eHealth Society, Warsaw, Poland. ^3^Center of Excellence "TeleOrto" for Telediagnostics and Treatment of Disorders and Injuries of the Locomotor System, Medical University of Warsaw, Warsaw, Poland. ^4^Department of Medical Informatics and Telemedicine, Medical University of Warsaw, Warsaw, Poland. ^5^Gabinet Lekarski, Warsaw, Poland

###### Correspondence: Wojciech Glinkowski

w.glinkowski@gmail.com

*Journal of Patient-Reported Outcomes* 5(1): O31.

**Objective**: The translation process is a complex, multi-stage, coordinated process in which many participants actively participate following the FACIT methodology. The authors aimed to develop an IT solution supporting the translation process and its effectiveness and efficiency.

**Methods: **The authors focused on supporting translating and creating the Translation Item History (TIH). The electronic TIH allows omitting sending back and forth spreadsheet files and speeds up the team's work. Each Translation Team Member (TTM) is considered a registered user since the platform enhances team formation and monitoring. IT tools used to build up the application consisted of an open-source database engine—PostgreSQL; Spring—an open-source backend framework for Java; Angular—open-source web application framework.

**Results:** The PROMIS translation platform becomes the only place to perform the translation tasks, and at the same time, it tracks and organizes every step of the translation process. The platform facilitates supervision over the process. Items are assigned to appropriate TTMs with their roles during the process steps. The user logs in, selects the correct translation, fills in a simple form, and carries out the task according to the protocol. The platform notifies each TTM response and prompts another active TTM to the following task. TTMs can observe all tasks in the dashboard as the list of issues to be done. Introduced mechanism enhances the performance of multiple translations simultaneously and speeds up the process. The system does not allow access to the translated text or unauthorized changes to it. The application generates and stores the Item Translation History as a spreadsheet and other necessary files about the process. Its use allows obtaining the final questionnaire in a standardized form as a PDF file. The final translation of items in Polish may have a different meaning than previously developed. The platform allows the use of complementary explanations of the meaning of Items if available.

**Conclusions:** The translation platform application based on the open-source solutions may serve as a supportive tool for obtaining the final versions of the verified quality translations with supervised creation of ITH

## 32* Intentionally omitted

### P33 Concurrent validity of PROMIS and SOSG-OQ in metastatic spine disease

#### Michelle Richardson^1^, David Bernstein^2^, Addisu Mesfin^3,1^

##### ^1^University of Rochester School of Medicine & Dentistry, Rochester, MN, USA. ^2^Massachusetts General Hospital, Harvard Combined Orthopaedic Residency Program, Boston, MA, USA. ^3^University of Rochester Medical Center, Rochester, MN, USA

###### Correspondence: Michelle Richardson

michelle96sfs@gmail.com

*Journal of Patient-Reported Outcomes* 5(1): P33.

**Objective:** While there remains a significant movement towards incorporating PROMs (patient-reported outcome measures) into clinical spine practice to improve patient-centered care, a commonly used PROM in practice remains elusive. Although the SOSG-OQ (Spine Oncology Study Group-Outcomes Questionnaire) was designed and validated for metastatic spine tumor patients, the use of general symptom-based PROMs, such as PROMIS (Patient-Reported Outcomes Measurement Information System) domains, may reduce both patient and physician burden and improve interdisciplinary care if shown to be concurrently valid.

**Methods:** Metastatic spine tumor patients from 1/2017 to 4/2021 at a single academic medical center were asked to complete PROMIS PF (Physical Function), PI (Pain Interference), and Depression domains and the SOSG-OQ. Only patients who completed both the SOSG-OQ and PROMIS instruments were included in the analysis. Spearman correlation (ρ) coefficients were calculated. Patients missing a single question in the SOSG-OQ were excluded from the correlation analysis of the corresponding section.

**Results:** A total of 87 unique visits, representing 67 patients met our inclusion criteria. A majority were men (50; 57%) and Caucasian (78; 90%), and the average age was 64 years (range: 34–87). There were 12 different types of tumors reported, with multiple myeloma, breast cancer, and prostate cancer representing 24 (28%), 22 (25%), and 11 (13%), respectively. Additional cancers included lung, colon, renal cell, thyroid, esophageal, non-Hodgkin’s lymphoma, large B cell lymphoma, plasmacytoma, and metastatic spindle cell sarcoma. SOSG-OQ was strongly correlated with PROMIS PI (ρ = 0.83) and moderately correlated with both PROMIS PF (ρ = 0.75) and PROMIS Depression (ρ = 0.57).

**Conclusions:** PROMIS PF, PI, and Depression appear to capture similar clinical insight as the SOSG-OQ. Spine surgeons can consider using these PROMIS domains in lieu of the SOSG-OQ in metastatic spine tumor patients.

### P34 Impact of COVID-19/social distancing on PROMIS pediatric peer relationship T-Scores in children with Legg-Calve-Perthes Disease

#### Ruchita Iyer^1^, Molly F McGuire^1^, Angel A Valencia^2^, Terri Beckwith^1^, Chan-Hee Jo^1^

##### ^1^Scottish Rite for Children, Dallas, TX, USA. ^2^UT Southwestern Medical Center, Dallas, TX, USA

###### Correspondence: Ruchita Iyer

Ruchita.iyer@utsouthwestern.edu

*Journal of Patient-Reported Outcomes* 5(1): P34.

**Objective:** The COVID-19 pandemic and subsequent social isolation can impact peer relationships in children, especially those with chronic disease. To assess the impact of social distancing in children with Legg-Calve-Perthes Disease (LCPD), we examined changes in the PROMIS Pediatric Short Form v1.0 – Peer Relationships 8a assessment before and during the pandemic.

**Methods:** Under retrospective IRB protocol, we identified 543 patients diagnosed with unilateral or bilateral LCPD. Due to differing county quarantine guidelines, geographic location was identified as a moderating construct. Using 2010 US Census Urban/Rural designations by county, patients’ zip codes were categorized as Urbanized Areas (population > 50,000), Urban Clusters (2,500–50,000), Rural (< 2,500) or International. Race, gender, age at first survey, and PROMIS T-scores were also collected by chart review. Operative events were considered moderating factors due to the potential for social interaction. Patients without visits between March 2019-March 2021 or missing/incomplete PROMIS were excluded.

**Results:** The Peer Relationship assessment was completed twice by 40 participants within March 1, 2020 ± 1 year. The average age was 11.3 years (7.8–17.7). Those who had surgery (80%), those who were male (75%), and those who were Caucasian (90%) had higher T-scores irrespective of time point. However, T-scores were similar across population densities (range 52.99–53.33). The average pre-pandemic T-score was 53.15; average during-pandemic T-score was 53.08 (*P* = 0.914). T-scores decreased for 32.5% of children (n = 13, average age 10.8 years), did not change for 22.5% (n = 9, average age 11.8 years), and increased for 45% (n = 18, average age 11.5 years). There was no statistically significant difference in change in T-score by race (*P* = 0.902), gender (*P* = 0.524), surgery (*P* = 0.266), or geographic location (*P* = 0.651).

**Conclusions:** Variables like race, gender, and surgical rates seemed to influence Peer Relationship scores in LCPD children but did not significantly influence changes to T-scores pre- and during-pandemic. Looking beyond the individual child and towards the community-level impact of the pandemic, children found ways to maintain their friendships despite COVID-19 restrictions, showing promise in their resiliency. Further study should seek to include presence of siblings as a source of peer influence, school district schedules, and individual experience of COVID-19 requiring social isolation.

### O35 Psychometric properties and reference values of PROMIS Fatigue item bank in the Dutch general population

#### Caroline Terwee^1^, Ellen Elsman^1^, Leo Roorda^2^

##### ^1^Amsterdam UMC, Vrije Universiteit, Amsterdam, Netherlands. ^2^Amsterdam Rehabilitation Research Center | Reade, Amsterdam, Netherlands

###### Correspondence: Caroline Terwee

cb.terwee@amsterdamumc.nl

*Journal of Patient-Reported Outcomes* 5(1): O35.

**Objective:** To assess the psychometric properties structural validity, measurement invariance / cross-cultural validity, and (internal) reliability of the Dutch-Flemish version of the v1.0 PROMIS Fatigue item bank in a general Dutch population and to assess Dutch reference values.

**Methods:** A representative sample of the Dutch general population from an internet panel completed the full V1.0 PROMIS Fatigue item bank. For structural validity we checked model assumptions (unidimensionality, local independence, monotonicity), fitted a Graded Response Model (GRM) and tested item fit. We evaluated measurement invariance by testing differential item functioning (DIF) for age, gender, education, region, and ethnicity. We assessed cross-cultural validity by testing DIF for language, comparing our sample to the US general population sample (n = 21.133) that was used for developing the item bank. T-scores were calculated for the full item bank, short forms (4a, 6a, 8a, and 7a) and a simulated CAT, using the US item parameters. We calculated the number of participants with a reliable T-score (SE < 3.16) for all measures. We calculated mean (SD) T-scores for the whole sample and age-range and gender subpopulations, and thresholds for mild, moderate and severe fatigue (0.5*SD, 1*SD, 2*SD).

**Results:** We included 1006 individuals (mean age 52 (SD 17), 53% female)). All assumptions were met (ECV 0.86, Omega-H 0.92) and all items fitted the GRM (item thresholds − 2.54–3.35, discrimination parameters 1.2–4.1). No items showed DIF for age, gender, education, region or ethnicity, but 7 items showed uniform DIF for language. In total, 98.3% of the respondents had reliable T-scores with the full item bank, 69.8 to 82.6% with the short forms, and 96.5% with the CAT (mean 5 items). Mean T-score of the Dutch general population was 49.1 (10.8). Thresholds for mild, moderate and severe fatigue were set to 55, 60, and 70, comparable to the US thresholds.

**Conclusions:** The PROMIS Fatigue item bank showed good psychometric properties, although DIF for language needs further study. Standardized measurement of fatigue using PROMIS across medical conditions will facilitate implementation and use of PROMs in clinical practice to support shared decision making and health care evaluation.

### P36 Femoral head deformity correlates with physical and mental health measures in healed Stage Legg-Calve-Perthes disease

#### Angel A. Valencia BS^1^, Chan-Hee Jo PhD^2^, Harry K. W. Kim MD^2,3^

##### ^1^University of Texas Southwestern Medical School, Dallas, TX, USA. ^2^Center for Excellence in Hip, Scottish Rite for Children, Dallas, TX, USA. ^3^Department of Orthopaedic Surgery, University of Texas Southwestern Medical Center, Dallas, TX, USA

###### Correspondence: Angel A. Valencia

angel.valencia@utsouthwestern.edu

*Journal of Patient-Reported Outcomes* 5(1): P36.

**Objective:** Legg-Calve-Perthes disease (LCPD) is a childhood ischemic osteonecrosis that can produce variable amount of femoral head deformity and progression from active to healed stage of the disease. Little is known about how the severity of deformity correlates with patient-reported quality of life measures at the healed stage of LCPD. The purpose of this study was to determine if the severity of femoral head deformity correlates with the Patient-Reported Outcomes Measurement Information System (PROMIS) Physical, Mental, and Social health measures at the healed stage.

**Methods:** We retrospectively analyzed 62 patients (45 male, 17 female) from a single institution who met the following eligibility criteria: unilateral LCPD in the healed stage, age > 11 (i.e. adolescent or older), and completion of 6 PROMIS Pediatric Short Form v2.0 measures: Mobility 8a, Pain Interference 8a, Fatigue 10a, Anxiety 8a, Depressive Symptoms 8a, and Peer Relationships 8a. We excluded patients who had surgery within 2 years of the survey. We used a continuous femoral head deformity score called Spherical Deviation Score (SDS) to assess the deformity on X-rays. Statistical analyses included Spearman’s Correlation to assess the relationship between the deformity and the PROMIS measures, and sub-analysis for age, gender, BMI, and history of surgery. ICC for intra-rater reliability of SDS measurements was also performed.

**Results:** 62 patients had a mean age at time of diagnosis of 7.9 ± 2.7 years (range 2–14.4) and a mean age at the time of survey of 14.4 ± 2.3 years (range 11–21). We observed significant correlation between the deformity (SDS) and patient-reported mobility (r = − 0.4 p = 0.002), pain interference (r = 0.3 p = 0.009), fatigue (r = 0.3 p = 0.01), anxiety (r = 0.5 p < 0.001), and depressive symptoms (r = 0.4 p < 0.001). No significant correlation was observed between the deformity and peer relationships (p = 0.3). SDS measurements showed excellent intra-rater reliability (ICC = 0.92).

**Conclusions:** Femoral head deformity correlated significantly with PROMIS physical (mobility, fatigue, pain interference) and mental health (anxiety, depressive symptoms) measures but not social health measure (peer relationships). These findings are clinically relevant as the severity of femoral head deformity is associated with patient-reported anxiety and depressive symptoms in LCPD.

### O37 Iterative testing of alternate stopping rules for the NIH Toolbox Emotion Battery Pediatric Item Banks

#### Saki Amagai, Rina Fox, Aaron Kaat, Michael Kallen, Benjamin Schalet, Cindy Nowinski, Richard Gershon

##### Northwestern University Feinberg School of Medicine, Chicago, IL, USA

###### Correspondence: Saki Amagai

saki.amagai@northwestern.edu

*Journal of Patient-Reported Outcomes* 5(1): O37.

**Objective:** Many self-report measures including the NIH Toolbox Emotion Battery (NIHTB-EB) and PROMIS seek to maximize precision while balancing burden. Although the current stopping rules for the computer adaptive testing (CAT) administration of these assessments are effective for some test takers, they can be burdensome for high-functioning individuals. Simultaneously, they yield inadequate reliability for some clinical populations. We evaluated four potential CAT stopping rules to increase reliability while minimizing burden.

**Methods:** We conducted simulations for general and clinical pediatric samples using 17 NIHTB-EB item banks, three of which are equivalent to PROMIS banks. The current CAT stopping rules terminate the test if ≥ four items have been administered, the standard error (SE) of the EAP score estimate is < 0.3, or a maximum of 12 items have been administered. The simulations considered the addition of a Standard Error (SE)-change rule (SE threshold for interim stopping reduced to 0.224) and a reduction of the maximum number of items, as well as examination of six- and eight-item fixed-length CATs. Simulees were grouped by the number of items administered and reliability achieved by each set of rules [Reliability < 0.85, 0.85 Reliability < 0.90, 0.90 Reliability < 0.95, 0.95 Reliability].

**Results**: Relative to the current rules, the SE-change rule minimally reduced average response burden (− 0.59 items general, − 1.2 items clinical). Although this rule increased the proportion of simulations achieving empirical reliability > 0.95 (+ 8.2% general, + 9.6% clinical), the average percentage of simulations achieving empirical reliability < 0.85 reached an unacceptable level (34.4% general, 20.9% clinical). Similarly, empirical reliability > 0.95 increased for six-item (+ 1.1% general, + 2.5% clinical) CAT; however, the percentage of simulations achieving reliability < 0.85 was excessively high (43.9% general, 31.4% clinical). Conversely, neither the eight-item CAT nor the reduced-maximum rule increased reliability < 0.85 relative to the current rules (eight-item: + 4.2% general, + 3.5% clinical; reduced-maximum: + 4.2% general + 3.6% clinical). The reduced-maximum rule also minimized burden by not always administering eight items (7.22 items general, 6.99 items clinical).

**Conclusions:** Each condition has potential advantages and disadvantages for specific research and clinical uses. We determined that the reduced maximum rule best balanced burden and precision for combined research and/or clinical use.

### O38 Optimizing the efficiency of computerized adaptive tests using real data: a machine learning approach.

#### Michiel Luijten^1,2^, Benjamin Schalet^3^, Leo Roorda^4^, Martha Grootenhuis^5^, Lotte Haverman^1^, Caroline Terwee^2^

##### ^1^Department of Child and Adolescent Psychiatry & Psychosocial Care, Emma Children's Hospital, Amsterdam UMC, Amsterdam, Netherlands. ^2^Department of Epidemiology and Data Science, Amsterdam UMC, Amsterdam, Netherlands. ^3^Department of Medical Social Sciences, Feinberg School of Medicine, Northwestern University, Chicago, IL, USA. ^4^Amsterdam Rehabilitation Research Center | Reade, Amsterdam, Netherlands. ^5^Princess Máxima Center for Pediatric Oncology, Utrecht, Netherlands

###### Correspondence: Michiel Luijten

m.luijten@amsterdamumc.nl

*Journal of Patient-Reported Outcomes* 5(1): O38.

**Objective:** To reduce administrative burden and increase efficiency of Computerized Adaptive Tests (CAT) our objective is to develop an additional stopping rule for CATs based on change in SE of a person’s estimated score (θ) after each administered item.

**Methods:** In April 2020 and November 2020 Patient-Reported Outcomes Measurement Information System (PROMIS) CATs were administered (*n-*range: 3212–3429). The stopping rules consisted of a standard error of measurement (SE(θ)) ≤ 0.32; 90% reliability) or a maximum of 12 items administered. Data on item selection, item responses, θ and SE(θ) of each step within the CAT were extracted. Datasplit into a training/test set. Using a machine-learning procedure the efficiency ((1- SE(θ)^2^)/n_items_) was maximized against the change in SE(θ) in the training set to determine the optimal change in SE(θ) to be used as a stopping rule. This stopping rule was subsequently applied to the test set (in addition to standard stopping rules) and the amount of participants reliably estimated (SE(θ) ≤ 0.32), average test length and relative efficiency were compared to using only the original stopping parameters. We applied this procedure to Anxiety (low α parameters) and Depressive Symptoms (high α parameters) CATs, as it is likely that the optimal stopping rule of change in SE(θ) is influenced by the discrimination parameters within the item response theory (IRT) model.

**Results:** Preliminary results show that on Depressive Symptoms/Anxiety, respectively 1193(35.9%)/1369(39.7%) of participants had 12 items administrated of which 33.9%/18.9% were due to floor effects. For these floor effects a change in SE of 0.01 would reduce the amount of items administrated from 12(SE(θ) = 0.588/(SE(θ) = 0.566)) to 4(SE(θ) = 0.619)/5(SE(θ) = 0.592), which results in a difference of T-score estimates of 3.3(32.0vs35.3)/ 3.4(31.9vs34.3).

**Conclusions:** Optimizing CAT efficiency by adding an additional stopping rule based on the change in SE(θ), may reduce the burden of PROMIS administration, while retaining precise, reliable measurements. Further optimizing the stopping rule will likely result in better trade-offs with fewer negative consequences to T-score estimates.

### P39 Advances in measuring mental health in early childhood (1–5 years): the PROMIS early childhood measures

#### Michiel Luijten^1,2^, Marthe Egberts^3^, Willemijn van Eldik^3^, Martha Grootenhuis^4^, Raphaële van Litsenburg^5^, Trudy Mooren^3^, Caroline Terwee^2^, Lotte Haverman^1^

##### ^1^Child and Adolescent Psychiatry & Psychosocial Care, Emma Children's Hospital, Amsterdam UMC, Amsterdam, Netherlands. ^2^Department of Epidemiology and Data Science, Amsterdam UMC, Amsterdam, Netherlands. ^3^Department of Clinical Psychology, Utrecht University, Utrecht, Netherlands. ^4^Princess Máxima Center for Pediatric Oncology, Utrrecht, Netherlands. ^5^Princess Máxima Center for Pediatric Oncology, Utrecht, Netherlands

###### Correspondence: Michiel Luijten

m.luijten@amsterdamumc.nl

*Journal of Patient-Reported Outcomes* 5(1): P39.

**Objective:** The American Psychiatric Association (APA) recently selected new level-2 DSM-V instruments for monitoring with shorter administration times, consisting of several Patient-Reported Outcomes Measurement Information System (PROMIS) measures for adults (aged 18 +) and children and adolescents (aged 8–18). To increase the age range of these measures the PROMIS initiative developed Early Childhood measures for measuring mental health in very young children (aged 1–5). These measures were translated to Dutch by forward and backward translations and cognitive debriefing. The objective of this study is to investigate the psychometric properties of four recently (2019) developed PROMIS Early Childhood (PROMIS EC) measures for assessing Anxiety, Depression, Irritability (anger) and Sleep Disturbance in the Dutch general population.

**Methods:** Secondary data analyses will be performed on data collected in 2020 – 2021 from a study that assessed the consequences of the COVID-19 outbreak on very young children. The Anxiety, Depression, Irritability and Sleep Disturbance complete item banks will be administered to parents of young (aged 1–5) children (n =  ~ 1300). To assess structural validity of each item bank a graded response model (GRM) will be fitted to the data after assessing the following assumptions: Unidimensionality through CFA (CFI > 0.95, TLI > 0.95, RMSEA < 0.10), local independence by residual correlations (r < 0.20) and monotonicity by Mokken analysis (*H* > 0.50, *H*_*i*_ > 0.30). Item fit of the GRM models will be inspected with S-X^2^, where *p* < 0.001 indicates misfit. Additionally, percentage of participants reliably measured will be assessed using the standard error of measurement (SEM) < 0.32 as a criterion (which equals a reliability of 0.90). If possible, differential item functioning (DIF) analyses will be performed between the Dutch and U.S. model.

**Results:** Translations were successful. Validation results will be presented at the conference.

**Conclusions:** After initial validation of these item banks, they can be implemented as CAT within the Netherlands to provide new measures to reliably and validly assess mental health in very young children (aged 1–5).

### O40 PROMIS Global Health: potential utility as a screener to trigger construct-specific PROs in clinical care

#### Brittany Lapin, Irene Katzan

##### Cleveland Clinic, Cleveland, OH, USA

###### Correspondence: Brittany Lapin

lapinb@ccf.org

*Journal of Patient-Reported Outcomes* 5(1): O40.

**Objective**: Patient-reported measures of health-related quality of life (HRQOL) are collected across healthcare systems to track patient conditions, evaluate change over time, and inform health policy. Many systems additionally collect construct-specific patient-reported outcome measures (PROMs). As patients and clinicians are inundated with surveys and data, efforts should be made to tailor survey administration to individual patient needs. Our study evaluated the ability of utilizing items on a measure of HRQOL to identify patients who may require additional screening.

**Methods:** A cross-sectional study was conducted of patients who completed PROMIS Global Health (GH) as part of routine care in a large healthcare system from 1/1/2016–12/31/2018. Additional construct-specific surveys were also routinely collected in some clinical centers. Receiver operating characteristic analysis was used to identify optimal thresholds for PROMIS-GH items predicting clinically meaningful thresholds on construct-specific PROMs: PHQ-9 score ≥ 10, Neuro-QoL Cognitive Function, PROMIS Physical Function, and Social Role Satisfaction T-score < 40, PROMIS Anxiety, Fatigue, Sleep Disturbance, and Pain Interference T-score > 60.

**Results:** Patients completed 1,085,599 PROMIS-GH surveys, with between 8,832 (for Neuro-QoL cognitive function) and 182,000 (PHQ-9) additionally completing one of the above construct-specific surveys. Scores ≤ 3 on PROMIS-GH item 10 (emotional problems) had 94.7% sensitivity (area under the curve (AUC) 0.867) for identifying patients with meaningful anxiety on PROMIS Anxiety and 90.0% sensitivity (AUC 0.820) for identifying patients with moderate-severe depressive symptoms on PHQ-9. Similarly high sensitivity and AUC were demonstrated for PROMIS-GH items assessing mental and physical health, ability to carry out social and physical activities, fatigue, and pain to identify poor scores in their corresponding construct-specific PROMs. Expectedly, worst performance was seen with the PROMIS-GH fatigue item when used to screen for poor PROMIS Sleep Disturbance scores (sensitivity 83.8%, AUC 0.712).

**Conclusions:** Our study provides preliminary support for the ability of utilizing PROMIS Global Health items as screening tools to identify patients who would most benefit from additional construct-specific PROMs. Through directing PROMs to patients for whom they are most applicable, survey burden is reduced for the majority of patients, allowing a more efficient and targeted use of PROMs to improve healthcare decision-making.

### P41 Validity and reproducibility of psychosocial questionnaires in French-speaking university students

#### Shirin Panahi^1^, Samir Sangani^2^, Mushirah Hossenbaccus^2^, Vicky Drapeau^1^, Sara Ahmed^2^

##### ^1^Université Laval, Québec, Canada. ^2^McGill University, Montreal, Canada

###### Correspondence: Shirin Panahi

spanahi329@gmail.com

*Journal of Patient-Reported Outcomes* 5(1): P41.

**Objective:** Sustainable health, a comprehensive state of physical, mental and social well-being that is attained and maintained throughout life, is affected by numerous factors. These include diet quality, physical activity, sleep and physical, mental, and social health. Patient-Reported Outcomes Measurement Information System (PROMIS), a platform that includes valid, reliable and standardized questionnaires, may be used to identify and manage several of these factors. The objective of this study was to evaluate 1) the acceptability of using PROMIS measures in a University Wellness Program, and 2) the relationship between PROMIS physical, mental and social measures with lifestyle factors such as diet quality, physical activity and stress in the French-Canadian university community.

**Methods:** All students (n = 2000; native French-speaking) will complete questionnaires on a web-based platform. The psychosocial questionnaires are tailored to each individual through a computerized adaptive testing platform, which uses algorithms to adapt the questions presented to the individual according to the answers provided for each question. During this stage, questionnaires related to sociodemographics, diet quality, physical activity, stress and COVID-19 will also be completed to determine any associations with PROMIS measures. Acceptability will be measured using open-ended questions to students about the value of completing measures. Pearson's correlations between the PROMIS measures, Nutrient-Rich Foods (NRF9.3) Index, an indicator of diet quality, physical activity, stress and sleep will be performed.

**Results:** The study started later than expected due to COVID in April 2021 and results will be presented using data collected until October 2021. It is expected that the PROMIS questionnaires will provide good validity (moderate to high correlations; *r* > 0.5). Furthermore, it is anticipated that several PROMIS measures will be associated with lifestyle factors such as diet, physical activity and sleep.

**Conclusions:** These results will provide evidence for the possible benefits of using PROMIS measures in university students. PROMIS may be used to support the development of interventions in the framework of services provided by the university to help students take charge of their physical, social and mental health and well-being.

### P42 Utility of interpretable general additive models in predicting PROMIS Global Health scores in orthopedic patients

#### Mark Fontana^1,2^, Vinicius Antao^1^, Miranda So^1^, Catherine MacLean^1,2^

##### ^1^Hospital for Special Surgery, New York, NY, USA. ^2^Weill Cornell Medical College, New York, NY, USA

###### Correspondence: Mark Fontana

fontanam@hss.edu

*Journal of Patient-Reported Outcomes* 5(1): P42.

**Objective:** To evaluate the utility of explainable boosting machine models in understanding which features predict PROMIS Global Health scores and how.

**Methods:** Between January 2017 and February 2021, we collected the PROMIS Scale v1.2 – Global Health (PROMIS-10) on patients scheduled for orthopedic surgery in seven subspecialties through online surveys and phone calls. To predict PROMIS scores, we used an interpretable general additive model, called an explainable boosting machine, which allows for automatic inclusion of non-linear and interaction effects and produces transparent visual explanations of model behavior. Separate models were trained to predict PROMIS-10 physical and mental t-scores using demographics, language preference, type of insurance, surgeon, and surgery subspecialty. We split data into 80% for model training and 20% for performance evaluation. We reported the top 5 most important predictors for each model and evaluated overall performance based on R^2 and root mean squared error (RMSE). Partial dependency plots for top predictors were reviewed, describing the exact mechanics of model performance.

**Results:** Among 121,426 surgeries, 95,870 had completed surveys (78.9%), among which 76,696 were used for model training, and 19,174 were used for model testing. The physical score model achieved a RMSE of 6.88 and R^2 of 0.21. The mental score model achieved a RMSE of 7.48 and R^2 of 0.13. For both models, the top predictors were surgical subspecialty, BMI, sex, insurance type, and age. Partial dependency plots were also similar between models. Spine subspecialty predicted lowest scores, while upper extremity predicted highest scores. BMI showed an inverted U-shaped relationship, with the highest scores predicted for patients between 18 and 23 kg/m^2. Men had higher predicted scores than women. Predictions declined with age, but with a “flat” range between 65 and 80.

**Conclusions:** Although interpretable general additive models did not predict preoperative PROMIS-10 physical and mental scores among orthopedic patients with particularly high performance, visualization of underlying model mechanics and patterns facilitated identification of which characteristics were most important for prediction, and that those features were leveraged in often non-linear ways.

### O43 Parkinson’s Disease and medication management: the relationship of cognitive ability -perception vs performance

#### Patrick Tierney^1,2^, Olivia Kaczmarek^2^, Avtej Sethi^2^, Meera Doshi^2^, Barbara Bumstead^2^, Marijean Buhse^2^, Elissa Kravis^2^, Bhupinder Anand^2^, Myassar Zarif^2^, Mark Gudesblatt^2^

##### ^1^Zucker School of Medicine at Hosftra/Northwell, Hempstead, NY, USA. ^2^South Shore Neurologic Associates, P.C., Patchogue, NY, USA

###### Correspondence: Olivia Kaczmarek

okaczmarek@southshoreneurologic.com

*Journal of Patient-Reported Outcomes* 5(1): O43.

**Objective:** Parkinson’s disease, a neurological movement disorder traditionally characterized by motor disturbance (tremor, rigidity, gait disturbance), but also impacts cognitive function, independence and self-care. Symptom management-based pharmacotherapy is the most common intervention and medication regimens can be complicated, burdensome, and change with disease progression. Cognitive decline in people with Parkinson’s Disease (PwPD) may reduce the capacity for independent medication management. Clinicians and patient’s appreciation of cognitive abilities might be inaccurate. Accurate identification of cognitive function might enhance care and outcomes by employing appropriate medication adherence strategies in PwPD. This study identifies the relationship between patient-reported medication management capabilities and patient-reported metrics of cognition with quantitative measures of cognitive ability.

**Methods:** Retrospective review of data collected through routine care of PwPD that were evaluated by standardized validated multidimensional computerized cognitive assessment battery (CAB, NeuroTrax) and completed patient reported outcomes (PROs): PROMIS Self-Efficacy for Managing Medications and Treatments Short Form 4a (MM) and PROMIS Applied Cognition- Abilities- Short Form 4a (AC). Cognitive domains evaluated included: Global Cognitive Score (GCS), Memory (MEM), Executive Function (EF), Visual Spatial (VS), Information Processing (IP), Verbal Function (VF), Attention (ATT).

**Results:** 90 PwPD, 64% male, average age 73 ± 9 years. Significant correlations were determined by regression analysis with p < 0.05 for the following metrics: MM vs GCS (r^2^ = 0.41), MM vs AC (r^2^ = 0.29). 31% of PwPD sampled had low confidence in managing medications, with 37% of males and 21% of females having low confidence in managing medications.

**Conclusions:** Increasing cognitive impairment in PwPD was associated with less confidence in effective and safe self-management of medications. Quantified measures of cognitive performance were more strongly associated with self-medication management efficacy than were perception of cognitive abilities in PwPD. Additional risk factors for impaired medication management in PwPD include low GCS and male gender. CAB in conjunction with MM PRO can provide value added information in care of PwPD. Recognizing the need for and incorporating strategies to assure effective adherence to treatment regimens can enhance care in PwPD.

### P44 Parkinson’s Disease psychosocial illness impact: relation to patient reported anxiety, depression & sleep disturbance

#### Avtej Sethi, Olivia Kaczmarek, Gina Koch, Angelina Thotam, Barbara Bumstead, Marijean Buhse, Elissa Kravis, Bhupinder Anand, Myassar Zarif, Mark Gudesblatt.

##### South Shore Neurologic Associates, P.C., Patchogue, NY, USA

###### Correspondence: Olivia Kaczmarek

okaczmarek@southshoreneurologic.com

*Journal of Patient-Reported Outcomes* 5(1): P44.

**Objective:** Parkinson’s disease (PD), a neurological movement disorder traditionally characterized by motor impairment (tremor, rigidity, gait disturbance), but also impacts cognitive function, independence and mood. Symptom management-based pharmacotherapy is the most common focus of care for people with PD (PwPD). Improving treatment satisfaction should include awareness of the non-motor aspects of disease impact. This study explores the relation between patient reported anxiety, depression, and sleep disturbance and the PROMIS Psychosocial Illness Impact-Negative–Short Form 4a in people with Parkinson’s Disease.

**Methods:** Retrospective review of data collected through routine care of PwPD that included completing patient reported outcomes (PROs): PROMIS Psychosocial Illness Impact-Negative–Short Form 4a (PII), PROMIS Sleep Disturbance – Short Form 4a (SD), and Hospital Anxiety and Depression Scale (HADS) which includes sub scores for anxiety (HADS-A) and depression (HADS-D).

**Results:** 90 PwPD, 64% male, average age 73 ± 9. Significant correlations were determined by regression analysis with p < 0.05 for the following metrics: PII vs HADS Global (r2 = 0.42), PII vs HADS-A (r2 = 0.315), PII vs HADS-D (r2 = 0.42), and PII vs SD (r2 = 0.12). 30% of patients had high psychosocial illness impact (score of > 16).

**Conclusions:** Unrecognized psychosocial illness impact is common in PwPD. Depression, Anxiety, and Sleep Disturbance are additional factors that impact psychosocial illness in PwPD. Patient reported anxiety and depression (HADS) was closely related to PII, where depression had a larger effect than anxiety determined by the subscales. Sleep disturbance also related and contributed to the impact of the psychosocial illness, albeit not as strongly as either depression or anxiety. Accurate awareness of non-motor aspects PD impact and identification and addressing care needs related to the psychosocial impact of PD may enhance treatment interventions. Clinicians' ability to provide targeted patient centric care for PwPD might allow for proactive appropriate targeted opportunities to improve quality of life (QoL). Psychosocial impact may have a large influence on the QoL and thus recognizing and addressing these factors might enhance care for PwPD.

### P45 Relationship of physical ability in Multiple Sclerosis to patient reported meaning & purpose

#### Olivia Kaczmarek^1^, Avtej Sethi^1^, Emma Malone^1^, Jamol Mardonov^1^, Barbara Bumstead^1^, Marijean Buhse^1^, Jeffery Wilken^2^, Daniel Golan^3^, Myassar Zarif^1^, Mark Gudesblatt^1^

##### ^1^South Shore Neurologic Associates, P.C., Patchogue, NY, USA. ^2^Neuropsychological Associates, Farifax, VA, USA. ^3^Department of Neurology, Carmel Medical Center, Haifa, Israel

###### Correspondence: Olivia Kaczmarek

okaczmarek@southshoreneurologic.com

*Journal of Patient-Reported Outcomes* 5(1): P45.

**Objective:** Multiple Sclerosis (MS) is an autoimmune disease characterized by relapses, progression, physical disability and MRI changes. Increasing disease impact is associated with physical disability, cognitive impairment, psychological impact and impaired social functioning. Traditional approach to patient care in MS focuses on identifying and treating the physical symptoms of MS with Disease Modifying Therapies (DMT), however the relationship of this to the overall patient experience remains uncertain. Patient reported outcomes (PROs) evaluating psychological and social functioning may provide value added information to identify critical patient-centric aspects that impact quality of life (QoL) and allow unrecognized opportunities to enhance outcomes and satisfaction. This study explores the impact of psychological, social and physical functioning on meaning and purpose in people with Multiple Sclerosis (PwMS).

**Methods:** Retrospective chart review of data collected through routine care of PwMS that completed PROs including: PROMIS Meaning and Purpose- Short Form 4a (MP), Patient Determined Disease Steps (PDDS), Neuro-QoL Ability to Participate in Social Roles and Activities – Short Form (SR), and Hospital Anxiety and Depression Scale (HADS).

**Results:** 345 PwMS, 73% female, average age 50.6 ± 11.7 years. Significant correlations were determined by regression analysis with p < 0.01: MP&PDDS (r^2^ = 0.07), MP&HADS-A (r^2^ = 0.17), MP&HADS-D (r^2^ = 0.40), and MP&SR (r^2^ = 0.26).

**Conclusions:** Meaning and purpose in PwMS is more closely correlated to psychological or social factors, rather than the physical disability. PRO physical disability (PDDS) demonstrated subtle negative correlation with MP, but SR and HADS both had large effect on MP, indicating that social and psychological function in PwMS may be a large contributor to patient QoL than previously anticipated. Enhanced understanding of such impact, identifying those with such impact and addressing these needs might provide unique opportunities to improve care, outcomes and satisfaction.

### P46 Multiple Sclerosis: the relationship of cognitive impairment and self-efficacy of medication management

#### Jack Petroski^1^, Olivia Kaczmarek^1^, Avtej Sethi^1^, Fatima Khan^1^, Barbara Bumstead^1^, Marijean Buhse^1^, Myassar Zarif^1^, Jeffrey Wilken^2^, Daniel Golan^3^, Mark Gudesblatt^1^

##### ^1^South Shore Neurologic Associates, P.C., Patchogue, NY, USA. ^2^Neuropsychological Associates, Fairfax, VA, USA. ^3^Department of Neurology, Carmel Medical Center, Haifa, Israel

###### Correspondence: Olivia Kaczmarek

okaczmarek@southshoreneurologic.com

*Journal of Patient-Reported Outcomes* 5(1): P46.

**Objective:** Multiple Sclerosis (MS) is a chronic disease for which there are multiple disease modifying therapies and symptomatic medications. Impaired self-efficacy of medication management can result in sub-optimal outcomes. Cognitive impairment in people with MS (PwMS) can impact multiple cognitive domains (CD) to varying degrees and combinations. The relationship of cognitive impairment across multiple CD to medication management in PwMS remains uncertain. Impaired medication management might adversely impact well-being, as well as social and physical functioning. Improved awareness of patient centric impaired self-efficacy of medication management might provide proactive opportunities for intervention. This study explores the relationships between self-efficacy of managing medication and cognition function in PwMS.

**Methods:** Retrospective chart review of PwMS who underwent standardized multi-domain computerized cognitive testing (CAB, Ntrax) and completed patient reported outcomes (PRO) including PROMIS Self-Efficacy for Managing Medication and Treatments (MM-4). CAB includes 7 cognitive domains: memory (Mem), executive function (Exe), attention (Att), information processing speed (Inf), visual spatial (Vis), verbal function (Ver), motor skills (Mot) as well as a global cognitive summary score (GCS).

**Results:** 338 PwMS (74% female, age = 50.6 ± 11.7 years) Regression modeling showed the following relationships between MM-4: GCS (r^2^ = 0.16, p < 0.05), Mem (r^2^ = 0.01, p < 0.05), Exe (r^2^ = 0.32, p < 0.05), Vis (r^2^ = 0.05 p < 0.05), Ver (r^2^ = 0.07, p < 0.05), Att (r^2^ = 0.29, p < 0.05), Inf (r^2^ = 0.21, p < 0.05), Mot (r^2^ = 0.08 p < 0.05).

**Conclusions:** Increasing cognitive impairment is associated with worse self-efficacy for managing medication and treatment. Progressive impairment of specific CDs are associated with progressive impairment of MM-4. Executive function shows the most significant relationship with MM-4 followed by attention and information processing. Incorporation of CAB and MM-4 into routine care can provide value added patient centric information that might offer opportunities to enhance care and outcomes in PwMS.

### P47 PROMIS 29-CAT vs. PROMIS 29-SF: CATs scratch out another win!

#### Michael Kallen^1^, Janel Hanmer^2^, Michelle Langer^1^, Richard Gershon^1^

##### ^1^Northwestern University, Chicago, IL, USA. ^2^University of Pittsburgh, Pittsburgh, PA, USA

###### Correspondence: Michael Kallen

michael.kallen@northwestern.edu

*Journal of Patient-Reported Outcomes* 5(1): P47.

**Objective:** PROMIS 29 is a widely used health status profile for concise assessment of eight domains. *Physical Function, Anxiety, Depression, Fatigue, Sleep Disturbance, Ability to Participate: Social Roles/Activities,* and *Pain Interference* utilize fixed-length short forms (SFs); *Pain Intensity* is measured by a single item. We sought to determine if score characteristics would improve if, with response burden held constant, scores were obtained via 4-item computer adaptive test (CAT) administration rather than by 4-item SF.

**Methods:**Samples. We simulated 2 samples per domain: one clinical (N = 2000), with a mean 1 SD in the direction of worse health; one non-clinical (N = 2000), with mean = 0 (theta metric). Scores. For each sample, we estimated 4 scores: full bank (our gold standard); 4-item CAT; 4-item SF response pattern (RP); 4-item SF conversion table (CT). Evaluation. We compared CAT, RP, and CT score (1) ranges; (2) correlations with gold standard; (3) root mean square differences (RMSDs) vs. gold standard; (4) mean SEs, mean reliabilities; (5) clinical vs. non-clinical mean differences, Cohen’s D effect sizes; and (6) item exposure, reflecting content coverage.

**Results:** For *Anxiety*: (1) CAT score ranges (clinical/non-clinical = 38.6–83.7/36.1–82.6) were “better” (greater) than RP and CT (40.4–81.4). (2) CAT score correlations with gold standard (clinical/non-clinical = 0.97/0.96) were “better” (higher) than RP and CT (0.96/0.92). (3) CAT score RMSDs vs. gold standard (clinical/non-clinical = 2.33/2.69) were “better” (less) than RP (2.92/3.81) and CT (2.94/3.82). (4) CAT score mean SEs (clinical/non-clinical = 2.77/3.30) were “better” (less) than RP (3.11/4.05) and CT (3.14/4.08), and CAT score mean reliabilities (clinical/non-clinical = 0.92/0.89) were “better” (higher) than RP (0.90/0.84) and CT (0.90/0.83). (5) CAT score clinical vs. non-clinical mean difference and Cohen’s D effect size (9.97/1.04) were “better” (greater) than RP (8.93/0.92) and CT (8.92/0.92). (6) CAT score item exposures (clinical/non-clinical = 12/12 total items) were “better” (greater) than RP and CT (4 common items). Other domain results, to be presented, showed similar advantages for CAT-obtained PROMIS 29 scores.

**Conclusions:** PROMIS 29 domain score measurement characteristics were improved upon in all areas evaluated under CAT vs. SF administration, while maintaining identical response burden across administration mode. Improvements occurred with clinical samples and more significantly under “difficult” assessment conditions, i.e., when measuring non-clinical samples.

### O48 Using latent growth curve/growth mixture modeling to investigate symptom change in ambulatory oncology

#### Michael Kallen^1^, Frank Penedo^2^, David Cella^1^, Sofia Garcia^1^

##### ^1^Northwestern University, Chicago, IL, USA. ^2^University of Miami, Miami, FL, USA

###### Correspondence: Michael Kallen

michael.kallen@northwestern.edu

*Journal of Patient-Reported Outcomes* 5(1): O48.

**Objective:** The Robert H. Lurie Comprehensive Cancer Center symptom assessment included 5 Patient-Reported Outcome Measurement Information System (PROMIS) domains measured using computer adaptive tests (CATs): Anxiety, Depression, Fatigue, Pain Interference, and Physical Function. When completed longitudinally, these assessments provide personal and group-level symptom trajectories (improving, declining, static) that can inform clinical care and research. We describe symptom status and change in a cohort of oncology outpatients and identify subgroups by their trajectories.

**Methods:**Sample. Following initial implementation of the assessment in routine clinical care, a convenience sample of 141 patients completed baseline (T1) and 3 subsequent assessments (T2-T4), each separated by 30 days or more. Using latent growth curve modeling (LGCM), we estimated symptom trajectories per domain, determining individual patient starting values (intercepts) and change rates (slopes), then summarized them at the group level. With growth mixture modeling (GMM), we investigated intercept/slope variability, identifying whether a single group (class) or multiple classes better accounted for observed variability. When the preferred solution was multi-class, we re-estimated symptom trajectories per class and compared class symptom characteristics.

**Results:** For all symptoms, assuming population homogeneity, we estimated common-class T1 starting values and change rates. With Pain Interference, the T1 start value was T-score = 49.6; change was essentially static (slope = − 0.01). However, with GMM we identified 2 distinct pain-associated patient classes and re-estimated their unique pain intercept/slope values: Class 1 (n = 91) had better (43.8/0.21) vs. Class 2’s (n = 50) worse pain status (59.8/− 0.41). At T1, classes differed in pain status by 16.0 T-score points; by T4 this difference decreased but remained considerable (11.4 T-score points). We re-estimated class-specific intercept/slope values for other symptoms evaluated: Class 2 reported significantly worse anxiety, depression, fatigue, and physical function status at T1 through T4.

**Conclusions:** These data, collected in routine cancer care, present an exciting opportunity to evaluate longitudinal patient-reported symptoms across a priority set of health domains. LGCM and GMM offer flexible methods for longitudinally characterizing domain status and change. They can be applied to investigate patient classes by clinical factors (e.g., cancer type, time since diagnosis, intervention) and, given available data, classes might be described by demographic and clinical status.

### O49 Integrating patient reported outcome measures into hospital quality initiatives

#### Judith Baumhauer^1^, Allison McIntyre^2^

##### ^1^University of Rochester Medical Center, Rochester, NY, USA. ^2^University Of Rochester Medical Center, Rochester, NY, USA

###### Correspondence: Judith Baumhauer

judy_baumhauer@urmc.rochester.edu

*Journal of Patient-Reported Outcomes* 5(1): O49.

**Objective:** Patient Reported Outcomes (PRO) collected routinely in the clinical setting have allowed healthcare providers to quantitatively validate and track how patients are feeling and functioning with various treatment options. Barriers to the implementation and utilization of these PRO data include other provider requirements to complete potentially less impactful quality improvement programs for “credits” linked with board certification, maintenance of certification (MOC), yearly state licensing and regulatory agencies obligations and insurance malpractice costs. Using PRO collection as a quality improvement initiative to fulfill other requirements may help eliminate barriers, align workflow and provide incentives to improve healthcare.

**Methods:** Three annual QI programs (Maintenance of Certification (MOC) Part IV MOC Continuing Medical Education Credit (CME) and 15% Malpractice Insurance reduction incentive) were identified. Working with the QA and Safety Office, the QI office and the Center for Experiential Learning, a PRO QI program was established which required PRO collecting, viewing, sharing and interpreting in the clinic office setting. Physicians would report the challenges and benefits of PRO collection including how PROs were used in decision pathways over a 3-month period.

**Results:** Utilizing the Plan-Do-Study-Act QI cycle for testing change by developing, implementing, observing the results, and acting on what is learned by physicians, the one PRO QI initiative was accepted by all three programs. This resulted in awarding up to 20 *American Medical Association (AMA) Physician’s Recognition Award (PRA) Category 1 Credits™,* 10–20 MOC part IV credits through the portfolio project and a 15% Malpractice insurance reduction, when teamed with a specific malpractice video module. In the second year of the PRO QI initiative, 48 providers participated and expanded their knowledge about PRO while satisfying requirements for these three important QI initiatives. Department participation increased 82% in one year with representation from 11 different medical specialties.

**Conclusions:** Integrating PROs into quality initiatives will allow providers to get credit for PRO integration into clinical decision making workflow. This will incentivize and align patient care with provider requirements and result in cost and time savings for providers.

### P50 Test–retest reliability and responsiveness of three Dutch-Flemish PROMIS CATs in physical therapy patients in primary care

#### Erik-Jan Haan^1,2^, Caroline Terwee^3^, Harriet Wittink^1^, Philip Van der Wees^4^, Henri Kiers^1,2^

##### ^1^Research Group Lifestyle and Health, HU University of Applied Sciences Utrecht, Utrecht, Netherlands. ^2^Association for Quality in Physical Therapy (SKF), Zwolle, Netherlands. ^3^Department of Epidemiology and Data Science, Amsterdam UMC, Vrije Universiteit Amsterdam, Amsterdam Public Health Research Institute, Amsterdam, Netherlands. ^4^IQ Healthcare and Rehabilitation, Radboud University Medical Center, Radboud Institute for Health Sciences, Nijmegen, Netherlands

###### Correspondence: Erik-Jan Haan

ejahaan@gmail.com

*Journal of Patient-Reported Outcomes* 5(1): P50.

**Objective**: The Dutch-Flemish Patient Reported Outcomes Measurement Information System Physical functioning v1.2 (DF-PROMIS-PF), Upper extremity v2.0 (DF-PROMIS-UE) and Pain interference v1.1 (DF-PROMIS-PI) item banks have been translated and validated in clinical samples.

The aim of this study is to examine the test–retest reliability, measurement error (Smallest Detectable Change (SDC)), responsiveness, and Minimal Important Change (MIC) of the DF-PROMIS-PF, DF-PROMIS-UE and DF-PROMIS-PI item bank administered as Computerized Adaptive Test (CAT) in patients receiving physical therapy.

**Methods**: Adult (> 18 y) patients with musculoskeletal disorders of the lower back, neck or upper extremity from 8 primary care clinics will be included in the study. At admission (T0), a questionnaire with demographic and clinical characteristics, the PROMIS CATs and standard used legacy questionnaires (Quebec Back Pain Disability Scale (QBPDS) for low back pain, Neck Disability Index (NDI) for neck pain and Disability of the Shoulder Arm or Hand questionnaire (DASH) for upper extremity disorders) will be administered. After 3 to 14 days (T1), the PROMIS CATs and anchor questions that measure change on the construct will be administered. At discharge (T2) the PROMIS CATs, legacy questionnaires and anchor questions will be repeated. Patients will be classified as “unchanged”, “deteriorated” or “improved” based on the response on the anchor questions.

The test–retest reliability of each PROMIS CAT will be determined by calculating the Intraclass Correlation Coefficient (ICC_2,1_) of the PROMIS CAT T-scores for “unchanged” patients between T0 and T1. Standard Error of Measurement (SEM) and SDC will be calculated as parameters of measurement error: SEM_agreement_ = √(σ^2^_measurement_ + σ^2^_residual_) and SDC = 1.96x√2 × SEM.

Responsiveness will be determined by testing *a priory* described hypotheses of expected correlations between changes in PROMIS CAT scores and changes in legacy PROM scores. Responsiveness will be considered sufficient when at least 75% of the hypotheses will not be rejected. The MIC will be calculated using predictive modelling.

**Results:** We aim to include at least 150 participants for each disorder (low back, neck or upper extremity). Initial results will be presented at the conference.

**Conclusions:** This is the first study to examine the test–retest reliability and responsiveness of PROMIS CATs in primary care physical therapy in The Netherlands.

### O51 Obtaining reliable change scores in the postoperative period: a comprehensive investigation of PROMIS pain items

#### Alexander Obbarius^1,2^, Stefan Schneider^1^, Doerte Junghaenel^1^, Arthur Stone^1,3^

##### ^1^Dornsife Center for Self-Report Science, University of Southern California, Los Angeles, CA, USA. ^2^Department of Psychosomatic Medicine, Charité—Universitätsmedizin Berlin, Berlin, Germany. ^3^Department of Psychology, University of Southern California, Los Angeles, CA, USA

###### Correspondence: Alexander Obbarius

alexander.obbarius@charite.de

*Journal of Patient-Reported Outcomes* 5(1): O51.

**Objective:** The Patient-Reported Outcomes Measurement Information System (PROMIS®) has invested much effort in the development of self-report tools to enable the measurement of individuals with high reliability. Measuring an individual’s health status and symptoms reliably does not necessarily mean that *changes* in these symptoms are equally captured with high reliability. High levels of reliability for individual change, however, are essential for detecting individual trends over time, such as symptom recovery following surgery. Using PROMIS pain measures collected in a post-operative period, we examined three aspects that may contribute to reliable change scores: measurement frequency, test length, and static versus adaptive testing.

**Methods:** Over almost 3 weeks following hernia surgery, 98 male patients competed daily diary versions of PROMIS pain interference and pain behavior short-forms. Based on these data, post-hoc simulations were conducted with the aim of comparing the ability of different strategies to achieve high reliability (i.e., > 0.9). Our simulations varied a) the number of measurement occasions over the study period (sampling density), b) the number of items (test length), c) and the mode of administration (i.e., static short-form vs. computer-adaptive testing [CAT]). Using a growth-curve modeling approach, observed change scores were compared to the best approximation of "real" (i.e., latent) change.

**Results:** When all pain interference or pain behavior items from all days of the study period were used, observed change scores showed near perfect reliability (i.e., approaching 1.0). The number of items and the number of measurement occasions both contributed to the reliability of observed change scores. In contrast to previous findings, CAT administration was generally superior to short-forms in achieving high reliability.

**Conclusions:** Various factors influence the reliability of change scores, including the sampling density, test lengths, and mode of administration. Further research should aim at identifying items that are best-suited for measuring change and, if required, add these items to existing PROMIS item banks.

## 52* Intentionally omitted

### O53 PROMIS Physical Function severity is associated with physical therapy recommendations in primary care

#### Ryan Jacobson^1^, Robert Long^2^, Dan Kang^1^, Mark Amendola^2^, Jeff Houck^1^

##### ^1^George Fox University, Newberg, OR, USA. ^2^Samaritan Health Services, Lebanon, OR, USA

###### Correspondence: Ryan Jacobson

rjacobson@georgefox.edu

*Journal of Patient-Reported Outcomes* 5(1): O53.

**Objective:** Triage treatment by physical therapists is an evolving service to improve diagnosis and outcomes in primary care. A challenge for health systems is to document outcomes of this service across a population. A potential outcome of primary care physical therapy (PC-PT) is to improve physical function across a population. However, current models of utilization focus on diagnosis rather than patient needs, as defined by the PROMIS Physical Function measure. The purpose of this study was to examine the association of recommendations from PC-PT for further physical therapy in primary care patients with musculoskeletal problems.

**Methods:** Patient records from Jan 2021 to April 2021 were requested from an evolving database to assess PC-PT in primary care (n = 383). PC-PTs were trained to use the PROMIS PF computer adaptive measure at intake to quickly assess perceptions of physical function. Training included interpreting the PROMIS PF measure in addition to other diagnostic decisions. Initial analysis was univariate (i.e. chi-square), followed by logistic regression, the outcome for both was referral to further outpatient PT. The predictor variables included: PROMIS PF severity (Very Low PF (< 40), Low PF (40.1–50), or Above Average PF (> 50.1)), age, gender, acuity of symptoms (acute, subacute, chronic), and area of injury (spine, extremity, other).

**Results:** Of 383 patients, 301 had complete data on all noted variables. A total of 40.5% (122/301) were recommended for physical therapy by the PC-PT. Chi square analysis showed no significant associations between recommendations for PT with gender p = 0.46), acuity categories (p = 0.07), or area of injury (p = 0.09). However, there was a strong association of PT referral with PROMIS PF categories (p < 0.001). The logistic regression analysis showed that age (p = 0.04), acuity (p = 0.07) and PROMIS PF (p =  < 0.001) categories influenced the recommendation of further physical therapy by the PC-PT. The accuracy when these three variables were included in the model was 67.1%.

**Conclusions:** PC-PT decisions are consistent with patient needs as defined by the PROMIS PF measure severity when recommending further physical therapy services following a primary care visit with the PC-PT. To improve population health outcomes, specialized programs may be needed to address patient needs (i.e. low PF) in addition to specific diagnostic categories.

### O54 PROMIS measures in geriatric, cardiorespiratory, neurologic and orthopedic rehabilitation patient populations: a systematic review

#### Rehab Alhasani, Nowaz Syed, Zhen Lun Chen, Qinyang Du, Suzanne Tran, Judith Soicher, Sara Ahmed

##### McGill University, Montreal, Canada

###### Correspondence: Rehab Alhasani

rehab.alhasani@mail.mcgill.ca

*Journal of Patient-Reported Outcomes* 5(1): O54.

**Objective:** One of the most crucial pieces of information that a clinician can gather to guide optimal patient care is the patient’s own perception of their health, symptoms, and well-being. However, it is unclear to what extent PROMIS has been used within rehabilitation patient populations. In this context, the purpose of this systematic review was to provide a comprehensive overview of PROMIS measures used with rehabilitation populations that can be used to inform patient care and guide future evaluation of PROMIS measures. Specifically, the authors aimed to 1) evaluate publication trends of PROMIS in rehabilitation populations, 2) evaluate the measurement properties, feasibility, and interpretation of PROMIS measures in rehabilitation populations.

**Methods:** We conducted a systematic review following the PRISMA guidelines. Articles were identified in MEDLINE/PubMed, EMBASE and CINAHL. Articles were excluded if they were not published in English or French after the year of 2004, if they did not focus on the adult rehabilitation population (geriatric, cardiorespiratory, neurological, and orthopaedic populations), and if they did not address the psychometric properties. Four reviewers, divided into two teams, screened abstracts, reviewed full articles and extracted the necessary information for this synthesis. The COSMIN guidelines were used to summarize and assess the psychometric quality of the PROMIS measures.

**Results:** A Kappa > 0.7 was achieved between reviewers. From 153 initial articles, 41 articles met the inclusion criteria covering the PROMIS, Neuro-QoL SCI-QoL, TBI-QoL measurement systems, and 14 domains across four patient populations (chronic conditions, neurological, orthopedic and geriatric). The most studied measures were the PROMIS Physical Function Computer Adaptive Test (PF CAT) (14 papers), followed by the PROMIS Pain Interference Computer Adaptive Test (PI CAT) (5 papers). Psychometric evaluations were most frequently reported for construct validity (70 instances), reliability (37 instances), responsiveness (17 instances) whereas content validity was reported the least (1 instance). Ratings of evidence for psychometric properties ranged from low to high across measures, property evaluated, and patient population.

**Conclusions:** The PF-CAT and PI-CAT were most commonly tested and had the strongest support for use in the orthopaedic population. Further research on PROMIS psychometric properties of other domains across populations is needed.

### P55 Common patient reported outcomes within the FDA Voice of the Patient Reports

#### Cameron Metz^1^, Polly McCracken^2^, Janel Hanmer^3^

##### ^1^University of Pittsburgh, School of Medicine, Pittsburgh, PA, USA. ^2^Department of General Internal Medicine, University of Pittsburgh, Pittsburgh, USA. ^3^Department of Internal Medicine, University of Pittsburgh, Pittsburgh, PA, USA

###### Correspondence: Cameron Metz

cad214@pitt.edu

*Journal of Patient-Reported Outcomes* 5(1): P55.

**Objective:** Proponents of disease-specific PROMs often argue that disease-agnostic measures do not adequately capture the experience of their patient population. PROMIS provides a set of disease-agnostic domains that may adequately cover many diseases. This study seeks to investigate if PROMIS’s quality of life domain coverage can span PROs across unrelated, chronic diseases as reported by patients.

**Methods:** The FDA’s Voice of the Patient Reports were an initiative located in the public domain to elevate patient voices regarding their condition and the associated therapies. Two reviewers independently extracted patient-reported health-related quality of life domains from the Reports and categorized them into PROMIS domains or non-PROMIS domains. For each report, the domain coverage was summarized. Any extracted PROs not covered by PROMIS domains were placed in an ‘other’ category and analyzed for common themes.

**Results:** Throughout all 26 reports, PROMIS covered 339 of 452 (75%) of the Reports’ domains. Sarcopenia and Heritable Bleeding Disorders tied for the highest coverage, 86%. HIV had the lowest coverage, 64%. The most common PROMIS domain, “Ability to Participate in Social Roles”, appeared in 25 (96%) reports. The least common PROMIS domain, itch, covered 5 (19%) reports. The most common domains not included in PROMIS were stigma, sensitivities, and sensory deficits as evident in 19 (73%), 18 (69%), and 18 (69%) reports, respectively. If the top three unincluded domains (stigma, sensitivities, and sensory deficits) were amended into PROMIS, the total domain coverage would increase from 75 to 94%.

**Conclusions:** PRO domains elicited in the FDA Voice of the Patient Reports were widely captured by PROMIS. This suggests the domains patients experience are similar enough to to be recorded by appropriate PROMIS domains. PROMIS could increase its coverage by adding domains for stigma, sensitivities, and sensory deficits. PROMIS remains a good candidate for the universal integration of PROMs.

### P56 Implementation of PROMIS CAT Pediatric Measures in outpatient behavioral health: adaptations and challenges during a pandemic

#### Kayla Hunt, Melissa Heatly, Linda Alpert-Gillis

##### University of Rochester School of Medicine & Dentistry, Rochester, NY, USA

###### Correspondence: Kayla Hunt

kmhunt2@gmail.com

*Journal of Patient-Reported Outcomes* 5(1): P56.

**Objective:** The purpose of this presentation is to discuss roll-out of the PROMIS CAT Pediatric and Parent Proxy measures during the COVID-19 crisis. Though we were scheduled to launch the PROMIS CAT measures via iPad during face-to-face visits in March 2020, we had to modify our original plan due to the onset of the pandemic. The launch was rescheduled for October 2020 with significant modifications for telehealth implementation; investigators collected feedback about the process from both families and clinicians. Presenters will discuss these modifications, strategies for adaptation, and reactions about the process obtained from families, youth, and clinicians.

**Methods:** The primary modification was the delivery method of the measures. While we were able to administer measures to some patients during face-to-face visits, the majority (80%) were seen via telehealth in October 2020. For telehealth visits, clinicians completed the measures with the patient/caregiver during the appointment, which took significantly more clinician time (10–30 min). Measures were administered to patients and caregivers via iPad during face-to-face visits. Patient/caregiver satisfactions surveys were distributed after completion of the measures. Clinician questionnaires assessed for attitudes, behaviors, and barriers related to utilization of standardized measures for both intake and progress monitoring and were administered pre-implementation and at 1, 3, and 6 months after implementation.

**Results:** Preliminary analyses included responses from 64 patients/caregivers and 38 clinicians. Overall, we received overwhelmingly positive responses from patients and caregivers about the process for completing the PROMIS CAT measures. Preliminary analyses of clinician questionnaires indicated that the administration time of measures was more of a barrier when completed via telehealth than when completed via iPad, which was consistent across time-points. Clinicians otherwise noted fewer barriers to implementation of measures over the six-month period. Future analyses will consider how implementation of the PROMIS measures are linked to changes in clinician attitudes about broader assessment and progress monitoring.

**Conclusions:** Despite the challenges we encountered during implementation during COVID-19, results indicate that the PROMIS CAT measures helped reduce barriers for use of standardized measures among clinicians, was an easier process for patients and caregivers, and will continue to help ensure that treatment our families receive high quality care.

### P57 Patient and caregiver satisfaction with PROMIS CAT Pediatric Measures in an outpatient behavioral health clinic

#### Kayla Hunt, Melissa Heatly, Linda Alpert-Gillis

##### University of Rochester School of Medicine & Dentistry, Rochester, NY, USA.

###### Correspondence: Kayla Hunt

kmhunt2@gmail.com

*Journal of Patient-Reported Outcomes* 5(1): P57.

**Objective:** The purpose of this presentation is to discuss implementation of a custom bundle of seven PROMIS CAT Pediatric and Parent Proxy Measures in a hospital-based outpatient behavioral health setting. Prior to implementation of PROMIS, lengthier paper-and-pencil measures were used for assessment and progress monitoring, which resulted in increased time burden for patients and caregivers and were not given at consistent time-points during treatment. During this roll-out, we obtained patient and caregiver feedback about their satisfaction with this new process.

**Methods:** Patients and caregivers were asked to complete the PROMIS measures during intake and then every 75 days during treatment. The seven measures included in the bundle were: Anxiety, Depressive Symptoms, Cognitive Function, Sleep Disturbance, Peer Relationships, Family Relationships, and Anger. Upon completion of those measures, patients and caregivers were asked to fill out a survey designed to assess satisfaction with computerized adaptive testing measures (see Krishna, Velleru, Smith, 2019). It includes six questions, each with a five-point Likert scale, regarding ease of use, completion time, relevance, utility, helpfulness, and willingness to repeat the measure again. We also asked whether the survey was completed by the child/teen or parent/caregiver and if it was completed during a face-to-face or telehealth visit. Data was collected via a web-based survey and remained separate from their EMR.

**Results:** Preliminary analyses included responses from 64 patients/caregivers. The majority of responses (94%) were collected during face-to-face visits and two-thirds of the respondents were parents/caregivers. Most responders (86%) found the program easy to use and were able to complete the measures in a reasonable amount of time (75%). They also reported they were asked relevant questions (84%) and would be willing to answer the questions again to track symptoms over time (78%). Responders indicated the information collected from these measures would be useful for the visit (72%) and their answers would help the clinician better understand their current challenges (72%).

**Conclusions:** Overall, we received positive responses from patients and caregivers about the process for completing the PROMIS CAT measures. Presenters will discuss next steps, limitations, and opportunities for future Quality Improvement efforts.

### O58 Turning data into information, information into insight and insight into action: data request system development

#### Allison W. McIntyre, Kathleen Fear, Judith Baumhauer

##### University of Rochester Medical Center, Rochester, NY, USA

###### Correspondence: Allison W. McIntyre

allisonw_mcintyre@urmc.rochester.edu

*Journal of Patient-Reported Outcomes* 5(1): O58.

**Objective:** Aggregate review of PRO data is necessary for clinical application and research and is only successful if there is access to robust datasets. However, it isn’t enough to have the data, it must be put to work.

Developing a standardized data request system that is nimble enough to adjust to the changing needs of the requestor, with access to data that was previously stuck in inaccessible silos, takes forethought. Only by planning ahead can systems be designed that are easy to use and manage and will produce data that can inform clinical decision making.

**Methods:** Transitioning from simple email requests to a standardized request process involved the use of a service desk software program. Once the single point of contact system was in place it was easier to collect required information, track requests and support regulatory requirements for research requests.

Consolidation was an important component of this project as data is pulled from multiple locations into one warehouse. Decisions about which components to include were based on previous data requests and review of similar systems across the enterprise.

Even with standardization, is often necessary to clarify requests. Having an integrated communication platform allows the analyst to exchange ideas, monitor changes and suggest tactics so the resulting data meets the needs of the requestor.

**Results:** Initial data requests were for administration metrics and patient PROMIS scores. After a year, with the introduction of monthly collection reporting, the majority of requests switched to longitudinal PROMIS and other PRO scores anchored by medical interventions or events. In 2020, 41 requests for data came through the system. 93% were for research or quality improvement initiatives and the rest for a variety of administrative evaluations. In the first quarter of 2021, all requests have been for research.

**Conclusions:** Clinical PRO data is typically not as clean as that collected as part of a research protocol. Having a standardized request system that guides the requestor and supports the data analyst is key to producing results that can yield new insight into how to improve clinical outcomes and value in healthcare.

### O59 Aligning significant individual change with patient-perceived meaningful change on the PROMIS Physical Function 10a

#### John Devin Peipert^1^, Ron Hays^2^, David Cella^1^

##### ^1^Northwestern University, Department of Medical Social Sciences, Chicago, IL, USA. ^2^UCLA, Department of Medicine/Division of General Internal Medicine & Health Services Research, Los Angeles, CA, USA

###### Correspondence: John Devin Peipert

john.peipert@northwestern.edu

*Journal of Patient-Reported Outcomes* 5(1): O59.

**Objective:** Individual change thresholds on patient reported outcomes (PRO) must be differentiable from error, or statistically significant. In addition, change should be meaningful to individuals. The reliable change index (RCI) identifies significant change but may be insensitive to patient-perceived meaningful change. An alternative is to relax the significance threshold for the RCI, creating a “likely change index” (LCI). We compared how RCI/LCIs categorized cancer patients as deteriorated with anchor-based methods on the physical function (PF) 10a Short Form.

**Methods:** In an observational, longitudinal study of 1129 adult cancer patients, the PF10a was given at baseline and 6 weeks later. A PF-specific patient global impression of change (PGIC) anchor categorized patients as deteriorated on PF (“a little worse” or “a lot worse”). We calculated an RCI (95% confidence) and LCIs (68% and 50% confidence). We estimated the group-level, anchor-based threshold for meaningful change using receiver operating characteristic (ROC) curve analysis. We compared how RCI/LCIs categorized patients as deteriorated with anchor-based thresholds (true condition) on the PF10a (individual threshold to group threshold comparison) and with the PGIC directly (individual threshold to individual threshold comparison).

**Results:** The ROC analysis suggested a threshold of -3 raw points. Agreement between the group-based threshold and the RCI/LCIs ranged between good [RCI 95% confidence: κ = 0.54] and perfect [LCI 50% confidence: κ = 1.00], and sensitivity increased as confidence level decreased. However, agreement between the PGIC anchor directly and RCI/LCIs always fell below the standard for “good” (κ < 0.40). Relaxing RCI/LCI to 68% and 50% confidence increased “false positives” (deteriorated on RCI/LCI but not on anchor) from 8 to 17% and 32%, respectively while “false negatives” (deteriorated on anchor but not on RCI/LCI) decreased as 22%, 17%, and 12%. Sensitivity and specificity were, respectively: RCI 95% = 0.40, 0.88; LCI 68% = 0.55, 0.73; LCI 50% = 0.68, 0.63.

**Conclusions:** Relaxing the significance threshold on the RCI increased its agreement with group-level anchor-based thresholds but not with an individual-level determination of meaningful deterioration. Since group-level thresholds average-out individual variation, more research is needed on how best to align significant and patient-rated meaningful change.

### P60 Implementing PROMIS Computerized Adaptive Tests in Systemic Lupus Erythematosus clinical care: patient and physician perspectives

#### Shanthini Kasturi^1^, Neena Patel^1^, Shreya Shetty^1^, Lisa Mandl^2,3^, Timothy McAlindon^1^, Amy LeClair^1^

##### ^1^Tufts Medical Center, Boston, MA, USA. ^2^Hospital for Special Surgery, New York,NY, USA. ^3^Weill Cornell Medicine, New York, NY, USA

###### Correspondence: Shanthini Kasturi

skasturi@tuftsmedicalcenter.org

*Journal of Patient-Reported Outcomes* 5(1): P60.

**Objective:** Patient-reported outcome measures (PROMs) are powerful tools that can facilitate person-centered care by highlighting individuals’ experience of illness. Little is known about the utility of implementing PROMs in the clinical care of patients with systemic lupus erythematosus (SLE), a chronic systemic autoimmune condition. This qualitative study aimed to evaluate the benefits and challenges of integrating PROMs into the routine clinical care of SLE from the perspective of patients and physicians participating in a multi-center longitudinal study.

**Methods:** SLE outpatients and treating rheumatologists participating in a longitudinal study of the implementation of PROMIS computerized adaptive tests in clinical care were invited to participate in focus groups and structured interviews. Focus groups of patients were conducted in-person and semi-structured interviews of physician were conducted via video teleconference. Patients and physicians were queried on the utility, benefits, challenges, and ideal implementation of PROMs in clinical care. All sessions were audio recorded and transcribed verbatim. Transcripts were reviewed to construct and refine a codebook using a comparison and consensus approach and a thematic analysis was performed.

**Results:** Twelve patients and 8 rheumatologists participated in focus groups and interviews. Patients and physicians reflected on the value of PROMs in facilitating communication and strengthening therapeutic relationships by highlighting and validating the patient experience of SLE. Patients found that PROMs enabled self-monitoring, but noted that the surveys were most useful when reviewed and discussed with their rheumatologists. Physicians believed PROMs promoted patient engagement and awareness, and emphasized their role in drawing attention to emotional health issues that might otherwise have been unaddressed. Both patients and physicians suggested that ideal clinical implementation of PROMs requires integration with the electronic health record, detailed guidance on score interpretation and population norms, and survey customization options.

**Conclusions:** SLE patients and rheumatologists participating in a longitudinal study of the implementation of PROMs in clinical care found that PROMs enhanced the care of SLE primarily by facilitating patient-physician communication and promoting patient self-reflection and validation. Optimal implementation of PROMs in routine SLE care requires physician engagement, easily interpretable scores, and integration with existing clinical platforms.

### O61 Implementation of PROMIS Computerized Adaptive Tests in Systemic Lupus Erythematosus (SLE) clinical care

#### Shanthini Kasturi^1^, Lori Lyn Price^1^, David Curtis^2^, W. Benjamin Nowell^2^, Norma Terrin^1^, Jane Salmon^3,4^, Lisa Mandl^3,4^, Timothy McAlindon^1^

##### ^1^Tufts Medical Center, Boston, MA, USA. ^2^Global Health Living Foundation, Upper Nyack, NY, NUSA. ^3^Hospital for Special Surgery, New York, NY, USA. ^4^Weill Cornell Medicine, New York, NY, USA

###### Correspondence: Shanthini Kasturi

skasturi@tuftsmedicalcenter.org

*Journal of Patient-Reported Outcomes* 5(1): O61.

**Objective:** Patient-reported outcome measures (PROMs) are powerful tools that can highlight the patient experience of illness. Although PROMs are standard metrics in SLE clinical research, they are not routinely integrated into the clinical care of this systemic condition. The aim of this study was to assess the feasibility and impact of implementing web-based PROMs in the routine clinical care of outpatients with SLE.

**Methods:** Outpatients fulfilling SLE classification criteria were enrolled in this longitudinal cohort study at two academic medical centers. Subjects completed PROMIS computerized adaptive tests at enrollment and prior to two consecutive routinely scheduled rheumatology visits using the ArthritisPower research registry mobile or web-based application. Score reports were shared with patients and providers before visits. Patients and rheumatologists completed post-visit surveys evaluating the utility of PROMs in the clinical encounters.

**Results:** A total of 105 SLE patients and 17 rheumatologists participated in the study. Subjects completed PROMs in 159 of 184 eligible encounters (86%, 95% CI 81 – 91) prior to study suspension due to the COVID-19 pandemic. Following baseline surveys, PROMs were completed for 90% (95% CI 82 – 95) of visit 1’s and 82% (95% CI 72 – 90) of visit 2’s. Nearly all PROMs (93%) were completed remotely. Patients and rheumatologists reported that PROMs were useful (91% and 83% of encounters respectively) and improved communication (86% and 72%). Rheumatologists found that PROMs impacted patient management in 51% of visits, primarily by guiding conversations (84%), but also by influencing medication changes (15%) and prompting referrals (10%). There was no statistically significant difference in visit length before (mean = 19.5 min) and after (mean = 20.4 min) implementation of PROMs (p = 0.52). Health-related quality of life and disease activity did not change significantly after implementation of PROMs, but patient activation improved in 14/23 (61%) of participants with low baseline activation levels.

**Conclusions:** The remote capture and subsequent integration of PROMs into clinical care was feasible in this diverse cohort of SLE outpatients. PROMs were useful to patients and rheumatologists, and promoted patient-centered care primarily by facilitating communication. Further studies are needed to clarify the impact of clinical integration of PROMs on activation and SLE-related outcomes.

### P62 Acceptability of PROMIS in immune-mediated Thrombotic Thrombocytopenic Purpura (iTTP): a mixed methods study

#### Deirdra Terrell^1^, Amanda Llaneza^1^, Rachel Kelley^1^, San Keller^2^, Sara Vesely^1^, James George^1^, Marshall Cheney^3^, Janna Journeycake^1^, Mohamad Khawandanah^1^, Adam Cuker^4^, Shruti Chaturvedi^5^, Neil Zakai^6^, Frank Akwaa^7^, Ming Lim^8^, Radhika Gangaraju^9^, Marshall Mazepa^10^, Spero Cataland^11^

##### ^1^University of Oklahoma Health Sciences Center, Oklahoma City, OK, USA. ^2^American Institutes for Research, Chapel Hill, USA. ^3^University of Oklahoma, Norman, OK, USA. ^4^University of Pennsylvania, Philadelphia, PA, USA. ^5^Johns Hopkins, Baltimore, MA, USA. ^6^University of Vermont, Vermont, VT, USA. ^7^University of Rochester, Rochester, NY, USA. ^8^University of Utah, Utah, UT, USA. ^9^University of Alabama at Birmingham, Birmingham, AL, USA. ^10^University of Minnesota, Minneapolis, MN, USA. ^11^Ohio State University, Columbus, OH, USA

###### Correspondence: Deirdra Terrell

dee-terrell@ouhsc.edu

*Journal of Patient-Reported Outcomes* 5(1): P62.

**Objective:** Immune-mediated thrombotic thrombocytopenic purpura (iTTP) is a rare disorder characterized by acute episodes of systemic microvascular thrombosis and thrombocytopenia. Focus groups of iTTP survivors revealed that persistent cognitive impairment, fatigue and depression/anxiety seriously impacts daily living even years after diagnosis. However, patient-reported outcomes are not systematically evaluated during long-term follow-up. The objective of this study was to determine acceptability and the preferred mode of administration of PROMIS instruments in iTTP. Understanding the patient preference will assist in integrating PROMIS instruments into clinical care.

**Methods:** Multi-center recruitment of survivors included: Oklahoma University, Ohio State University, University of Minnesota, Johns Hopkins University, University of Rochester, University of Pennsylvania, University of Alabama at Birmingham, University of Utah and the University of Vermont. Following informed consent, survivors were given PROMIS cognitive function ability, anxiety, and fatigue instruments via their preferred mode of administration. Descriptive statistics were used to summarize participant scores relative to the PROMIS normed mean score on each domain. Additionally, typical internet usage, behaviors regarding searching for health information online and demographics were obtained. Qualitative assessment regarding acceptability of these PROMIS instruments is ongoing (completed 37 of planned 45 interviews).

**Results:** To date, 88 survivors have completed the surveys (82% female; 49% White; 33% Black; median age 49.5 years (range 25–85 years)). 52% preferred completing PROMIS surveys online vs. self-administered or telephone administered. However, among Black survivors, 31% preferred online compared to telephone/self-administered. Overall, survivors report that the internet is helpful to find health information and to assist in understanding doctor’s instructions. iTTP survivors descriptively scored worse than the general population (mean 50; standard deviation 10) on all PROMIS domains. Lower scores illustrate worse function on cognitive function ability and higher scores illustrate worse function on anxiety and fatigue. Means (standard deviations) were as follows: cognitive function ability 45 (9); anxiety 59 (10); fatigue 58 (10).

**Conclusions:** iTTP survivors in remission have cognitive, fatigue and anxiety scores that illustrate worse function than the US general population. Overall, survivors preferred online administration. However, Black survivors preferred other administration methods. Recognizing these preferences is a vital next step toward integrating PROMIS into routine care.

### P63 Validation of PROMIS physical function item bank (CAT) in patients on kidney replacement therapies

#### Wajiha Ghazi, Evan Tang, Eric Mauti, Yingji Sun, Karma Gyatso, Rabail Siddiqui, Nasab El-Dassouki, Ward Hajjar, Anqi Chen, Aysha Afzal, Ghazaleh Ahmadzadeh, Istvan Mucsi

##### Multi-Organ Transplant Program and Division of Nephrology, University Health Network, Toronto, Canada

###### Correspondence: Wajiha Ghazi

wajihaghazi28@gmail.com

*Journal of Patient-Reported Outcomes* 5(1): P63.

**Objective:** Poor physical function (PF) is an independent risk factor for mortality among patients with chronic kidney disease. Here we validate the Patient Reported Outcome Measurement Information System (PROMIS) PF item bank administered using computer-adaptive test (CAT) among patients treated with kidney replacement therapies (dialysis or kidney transplant).

**Methods:** A cross-sectional sample of adults treated with kidney transplant or dialysis completed PROMIS PF CAT using tablet-based electronic data capture. Participants also completed a sociodemographic questionnaire and legacy questionnaires (12-item Medical Outcomes Study Short Form [SF-12] and the EQ-5D-5L). Clinical data was extracted from medical records. We assessed reliability on the participant level by standard errors of measurement (SEM) over the range of T-scores. SEMs were converted to reliability coefficients (1–SEM^2^). Average reliability was calculated as 1-∑(standard error)^2^. Test–retest reliability was assessed using the intraclass correlation coefficient (ICC) in a subgroup of participants who repeated PROMIS PF CAT within 3 to 14 days. Convergent validity was assessed using correlation between PROMIS PF CAT and SF-12 physical component summary (PCS). Discrimination was assessed using receiver operating characteristic (ROC) curves with the EQ-5D mobility item as a reference for “impaired mobility”, categorized as ‘no problems’ versus ‘any mobility problems’.

**Results:** Of the 371 participants, mean (SD) age was 56(16) years, 64% male and 46% White; 64% had received a kidney transplant whilst 36% were on dialysis. Mean (SD) hemoglobin level was 123(19) g/L. The mean (SD) PROMIS PF T-score was 44(11). The average reliability was high (0.94) with 98% of individual reliability coefficients > 0.90 over the T-score range of 15–66. Test–retest reliability was good (ICC = 0.91, n = 75). A strong correlation (Rho = 0.77) was observed between PROMIS PF T-score and SF-12-PCS. ROC analysis confirmed that PROMIS PF CAT had very good discrimination for “impaired mobility” (area under the ROC = 0.83). Overall similar results were seen in the dialysis and kidney transplant sub-cohorts.

**Conclusions:** Our results support the validity and reliability of PROMIS PF CAT among patients treated with kidney replacement therapies. PROMIS PF CAT may be useful to monitor physical function in clinical and research settings.

### P64 Association of PROMIS-Cognitive Function Scores with the Montreal Cognitive Assessment Test in a clinical sample

#### Maria Edelen^1,2^, Janel Hanmer^3^, Jin-Shei Lai^4^, Michelle Langer^4^, Anthony Rodriguez^2^, Beth Dana^2^

##### ^1^Brigham and Women's Hospital, Boston, MA, USA. ^2^RAND Corporation, Boston, MA, USA. ^3^University of Pittsburgh Medical Center, Pittsburgh, PA, USA. ^4^Northwestern University, Chicago, IL, USA

###### Correspondence: Maria Edelen

orlando@rand.org

*Journal of Patient-Reported Outcomes* 5(1): P64.

**Objective:** To assess the association of self-reported cognitive function using 2 PROMIS items to the clinician-administered Montreal Cognitive Assessment (MOCA) test in a clinical sample.

**Methods:** A large health system in western Pennsylvania began routine collection of 2 items from the PROMIS cognitive function item bank (PROMIS-CF) whenever a new patient-reported outcomes data collection was constructed for their electronic medical record (EMR) starting 12/2017. We extracted data from all patients with PROMIS-CF scores from the EMR, and examine the subset of patients who also had a MOCA assessment within 30 days of the PROMIS-CF. MOCA scores range from 0 to 30 with a score of 26 or higher considered normal. We hypothesize that 1) patients who have a recorded MOCA will have lower PROMIS-CF scores than those who do not, 2) the proportion of patients who have an abnormal MOCA score will increase as PROMIS-CF scores decrease, and 3) MOCA and PROMIS-CF scores will be positively correlated.

**Results:** PROMIS-CF was collected at least once in 50,820 people between 12/2017 and 3/2020. These data were collected primarily in Neurology clinics. Among patients who had a PROMIS-CF score, 1,303 had a MOCA score, with 75% of MOCAs administered on the same day as the PROMIS-CF and 25% within 30 days. The distribution of PROMIS-CF scores for all patients ranged from 29.7 to 62.2 with a mean of 48.9 and a median of 47.6. The distribution of PROMIS-CF scores for patients with a MOCA had the same range but a slightly lower mean (46.1) and median (44.0). The percentage of patients with an abnormal MOCA score increased with decreasing PROMIS-CF scores as hypothesized from 72% for patients with a PROMIS-CF score over 60, to 77% for scores 50–60, 79 for scores 40–50, and 89% for scores under 40. The MOCA and PROMIS-CF scores were only moderately correlated (*r* = 0.19) but that relationship increased to 0.25 when subset to patients completing both assessments on the same day.

**Conclusions:** These preliminary results indicate that a 2-item PROMIS-CF score may be clinically useful in identifying patients who need further cognitive function evaluation.

### P65 Patient and family caregiver perspectives on self-assessment cognitive screening in primary care

#### Julia Bandini^1^, Lucy Schulson^1^, Jordan Harrison^2^, Sangeeta Ahluwalia^3^, Maria Edelen^1,4^

##### ^1^RAND Corporation, Boston, MA, USA. ^2^RAND Corporation, Pittsburgh, PA, USA. ^3^RAND Corporation, Santa Monica, CA, USA. ^4^Brigham and Women's Hospital, Boston, MA, USA.

###### Correspondence: Lucy Schulson

schuls6on@rand.org

*Journal of Patient-Reported Outcomes* 5(1): P65.

**Objective:** To evaluate patient and family perspectives on cognitive screening through self assessment for early detection of cognitive impairment in primary care.

**Methods:** We conducted two virtual focus groups with 1) primary care patients age 65 and older and 2) family caregivers of patients with cognitive impairment/dementia. Participants included 18 patients and 5 family members. Participants were recruited from a large health system in western Pennsylvania. We used a structured guide to assess comfort answering questions for screening for cognitive impairment and preferences regarding mode of administration, types of screening questions, and follow-up for a positive screen.

**Results:** Patients expressed a general sense of comfort in answering questions around their memory. Patients preferred to have this conversation with a primary care provider (PCP) with whom they had an established relationship and were less comfortable answering questions if asked by other medical staff (e.g. a medical assistant). In terms of mode of screening, most patients preferred to complete a cognitive screener with their PCP during an appointment or in advance via online portal, as opposed to a mailed questionnaire or text message. Patients preferred screening questions that reflect specific actions rather than general debility. Patients expressed that they would want to be informed by their provider if experiencing any signs of cognitive decline and felt that follow-up actions are important.

The early signs of cognitive impairment that family caregivers noted in their loved ones were personality/mood changes, repetitive conversations, and difficulty adapting to new environments. Despite early warning signs, diagnosis of cognitive impairment/dementia often occurred after an acute episode such as a hospitalization not directly related to cognitive impairment. Caregivers expressed that early diagnosis may help facilitate planning for the future and felt it was critical for this process to begin with the PCP.

**Conclusions:** Preliminary findings suggest that self-assessment cognitive screener in primary care is acceptable to patients and family caregivers, though they emphasized the importance of having an established relationship with their PCP. Patients and caregivers preferred the PCP to have a central role in discussions around cognitive impairment, follow-up for a positive screen, and planning for the future.

### P66 Multistep translation and cultural adaptation of the Chinese version of PROMIS Item Bank-Psychosocial Illness Impact

#### Danruo Wang^1^, Ling Yuan^2^, Lijuan Bian^3^, Ligui Wu^3^, Rui Wang^4^

##### ^1^The Comprehensive Cancer Centre of Drum Tower Hospital, Medical School of Nanjing University, Nanjing, China. ^2^Department of Nursing of Drum Tower Hospital, Medical School of Nanjing University, Nanjing, Nanjing, China. ^3^The Comprehensive Cancer Centre of Drum Tower Hospital, Medical School of Nanjing University, Najing, China. ^4^The Comprehensive Cancer Centre of Drum Tower Hospital, Medical School of Nanjing University, Nanjin, China

###### Correspondence: Danruo Wang

wangdanruo@163.com

*Journal of Patient-Reported Outcomes* 5(1): P66.

**Objective:** In order to complete the translation and measurement test of the Chinese version of PROMIS, and to promote the clinical application of PROMIS in China, our team voluntarily applied to join the Chinese version of PROMIS translation organized by Patient-Reported Outcomes Measurement Information System National Center-China (PNC-China) under the authorization of PROMIS Health Organization (PHO).

**Methods:** We set up a professional team of 10 people to complete the translation work, which leaded by the translation project manager (TPM), a deputy director of the nursing department. In this team, there are 2 medical experts, who are also doctoral mentors, 1 linguist, 3 nurse specialists with master's degree, 3 nursing postgraduates. To translate and culturally adapt the PROMIS Item Bank v1.0-Psychosocial Illness Impact-Negative and Positive, we followed the international standard Functional Assessment of Chronic Illness Therapy (FACIT) translation method required by PROMIS data management center.

**Results:** After simultaneous forward translation and reconciliation conducted by 3 Chinese speaking researchers, and back translation conducted 1 English speaking researcher who is proficient in Chinese back translation, the TPM conducted a back translation review. In the expert review step, 2 medical experts and 1linguist selected the most appropriate translation for each item. After that, the TPM collates and analyzes the three expert opinions, evaluates the advantages and disadvantages of each opinion and formed problems and comments. Based on the pre finalization review, the language coordinator (LC), whose mother language is Chinese, selected the most appropriate translation after careful analysis, combining with the expert opinions and the opinions of the TPM, and then decided the final translation.

**Conclusions:** To complete the harmonization and quality assurance, the TPM submitted the back translation and the relevant opinions of the whole translation process to PNC-China and PROMIS data management center for quality evaluation. Further verification and application will be carried out after receiving the feedback massage from the PROMIS data management center.

### P67 Are biopsychosocial PROMIS measures associated with risk of persistent symptoms in outpatient physical therapy?

#### Jeff Houck^1^, Rebecca Dobler^1^, Chris Hoekstra^2^

##### ^1^SUNY Upstate Medical University, Syracuse, NY. ^2^Therapuetic Associates, Inc, Tualatin, OR, USA

###### Correspondence: Jeff Houck

HouckJ@upstate.edu

*Journal of Patient-Reported Outcomes* 5(1): P67.

**Objective:** The newly validated Keele STarT MSK Tool ^©^ (MSK Tool) stratifies primary care patients into low, medium or high risk of persistent musculoskeletal (MSK) pain. The multidimensional tool includes items for pain, pain coping, comorbidities, and function and disability. Health domain specific Patient Reported Outcomes Measurement Information System (PROMIS) outcomes representing a biopsychosocial patient experience may also predict risk of persistent pain. The purpose of this study was to report which PROMIS health domains influence the MSK Tool score, and therefore assist clinicians select the best outcomes associated with the risk of persistent symptoms.

**Methods:** From April 2020 to October 2020 patients (n = 94, age = 46.7(18.9) years, 67.4% female) attending two outpatient orthopedic clinics agreed to complete questionnaires during their physical therapy visit. PROMIS computer adaptive tests were collected concurrently with the MSK Tool. Seven PROMIS measures categorized as *physical* (physical function [PF], pain interference [PI], Fatigue), *psychological* (Anxiety [Ax], Depression [Dep], Self-Efficacy of symptom management [SE]) and *social* (Satisfaction with Social Roles [SR]) were completed. Three logistic regression models were considered. Model one included physical health domains, model two included only psychological domains and model three included all three domains.

**Results:** The MSK Tool average score was 5.1(2.5). The majority of patients had extremity problems (44.6%) followed by spine (29.7%). A total of 46.8% were low risk, 43.6% moderate risk and 9.6% high risk. Two of the *physical* measures, PI and Fatigue, were retained with an r^2 ^= 0.46. Standardized coefficients favored Fatigue (PI = 0.29 versus Fatigue = 0.46) as more influential. Two of the *psychological* measures, Dep and SE, were retained with an r^2 ^= 0.38. Standardized coefficients favored SE (Dep = 0.23 versus SE = − 0.45) as more influential. Three measures were retained when all measures were included (i.e. SE, PI and Fatigue) with an r^2^ = 0.49. Standardized coefficients favored Fatigue (Fatigue = 0.35 then PI = − 0.24 then SE = − 0.21) as more influential.

**Conclusions:** Risk of persistent MSK symptoms is highly associated with PI, Fatigue and SE of symptom management. Utilization of the PROMIS SE and Fatigue measures with pain interference should be considered for predicting patients at risk of persistent MSK symptoms.

### P68 Translation and linguistic validation of the PROMIS-29 + 2 Profile (PROPr) in seven African languages

#### Helena Correia^1^, Jiyoung Son^1^, Christina Dionesotes^2^, David Cella^1^

##### ^1^Northwestern University, Chicago, IL, USA. ^2^RWS Life Sciences, East Hartford, CT, USA

###### Correspondence: Helena Correia

helena-correia@northwestern.edu

*Journal of Patient-Reported Outcomes* 5(1): P68.

**Objective:** To translate and linguistically validate the PROMIS-29 + 2 Profile (PROPr) in seven African languages: Dholuo, Dhopodhola, Lusoga, Swahili, Teso and Twi. We will discuss the linguistic and cultural issues encountered during the harmonization across languages and cognitive debriefing in three countries.

**Methods:** Translations were conducted according to the PROMIS standards, through an iterative process of forward and back-translation, multiple reviews and cognitive debriefing. Testing took place in Uganda, Kenya, and Ghana with five native-speaking participants from each language. Participants completed the questionnaire and participated in a cognitive debriefing interview to verify understandability, relevance and appropriateness of the translations. Qualitative analyses of participants’ comments assessed the conceptual equivalence across languages.

**Results:** One major challenge was that some of the English source concepts were not well distinguished from each other in the target languages. For example, in Dhopodhola, Luo, and Luganda, there is no clear distinction between "anxiety" and "depression". Experiencing emotional distress is often expressed as "being in deep thought" and this expression covers various feelings in the emotional distress spectrum such as feeling "anxious", "depressed", "worried", or "fearful". "Fatigued" proved difficult to translate in all languages, because it is not distinguished from "tired" or "run-down". Discussion of alternatives and clarification of intended meaning were necessary in order to find suitable equivalent wording in each language. Cognitive testing indicated that most translated items were well-understood in each language. When participants' comments revealed misunderstanding of an item's intended meaning, the translation was revised to ensure cultural appropriateness, conceptual equivalence and harmonization across similar languages.

**Conclusions:** The PROMIS-29 + 2 (PROPr) is now available in Dholuo, Dhopodhola, Lusoga, Swahili, Teso and Twi. Given the item overlap, the PROMIS-29 profile and its associated short forms are also available in these languages.

### O69 Validation of the PROMIS physical function computer adaptive test among liver transplant recipients

#### Evan Tang^1^, Eric Mauti^1^, Nazia Selzner^2^, Ward Hajjar^2^, Nathaniel Edwards^2^, Istvan Mucsi^2^, Susan Bartlett^3^

##### ^1^Temerty Faculty of Medicine, University of Toronto, Toronto, Canada. ^2^Kidney Transplant Program, Ajmera Transplant Centre, University Health Network, Toronto, Canada. ^3^Centre for Health Outcomes Research, McGill University, Montreal, Canada

###### Correspondence: Evan Tang

evan.tang@mail.utoronto.ca

*Journal of Patient-Reported Outcomes* 5(1): O69.

**Objective**: Physical function (PF) integrates motor function, motor control, and physical fitness. PF is an important component of everyday functioning and health-related quality of life. The Patient Reported Outcomes Measurement Information System (PROMIS) computer adaptive test (CAT) may offer greater precision and lower response burden compared to legacy questionnaires. Here we validate the PROMIS PF item bank administered as CAT in liver transplant recipients (LTR).

**Methods:** A cross-sectional, convenience sample of adult LTR completed the PROMIS PF CAT, the 36-Item Short Form Health Survey (SF-36), and the EQ5D5L questionnaire using electronic data capture. Socio-demographic and clinical data were also collected. Reliability was assessed using average reliability, measurement error (standard error of measurement [SEM]), and test–retest reliability (intraclass correlation coefficient [ICC]). Construct validity was assessed using Pearson correlation between PROMIS PF and SF-36 PF scores, and known-group comparisons (higher scores expected in individuals without anemia, with fewer comorbidities, with better self-reported health). Discrimination of PROMIS PF CAT was assessed using receiver operating characteristic analysis. Reference categories (mobility problems yes/no) were defined by responses the mobility domain of the EQ5D5L (no problem vs. any problems).

**Results:** Our sample included 160 LTR with a mean(SD) age of 55(15), 69% male, and 66% white. 57 participants (36%) reported mobility problems. Participants completed a mode (range) of 4 (4–12) PROMIS PF CAT questions, and 97% (n = 151) had SEM < 0.30 (reliability > 0.90). Average reliability was good at 0.95 and ICC was 0.92. The PROMIS PF T score correlated strongly with SF-36 PF domain (r = 0.84). Construct validity was further confirmed by known-groups comparisons, as higher PF scores were reported in participants without anemia (Cohen’s d [d] = 0.73; p < 0.001), lower comorbidity burden (d = 0.60; p < 0.001), better self-reported health (d = 1.19; p < 0.001), and greater mobility (d = 1.52; p < 0.001). The PROMIS PF CAT showed good discrimination for impaired mobility, with a c-statistic of 0.87.

**Conclusions:** The data supports the reliability and validity of the PROMIS PF CAT for measuring PF in LTR.

### P70 Exploring the relationship between paediatric PROMIS profile-25 and a Kidscreen utility-mapping algorithm

#### John Chaplin^1^, Peter Adolfsson^2^

##### ^1^Sahlgrenska Academy at Gothenburg University, Gothenburg, Sweden. ^2^Department of Pediatrics, Kungsbacka Hospital, Kungsbacka, Sweden

###### Correspondence: John Chaplin

john.chaplin@gu.se

*Journal of Patient-Reported Outcomes* 5(1): P70.

**Objective:** Few cost-utility studies of child and adolescent health use quality-adjusted life years as the outcome. A recent study has built an algorithm between the Child Health Utility (CHU9D), a generic instrument developed specifically for use in young people, and the Kidscreen-10 index. The purpose of this study was to calculate the CHU9D scores based on Kidscreen and compare these to PROMIS profile-25 scores within the same population of young people to assess the predictive value.

**Methods:** This is a cross‐sectional study of children with type 1 diabetes, recruited from tertiary care facilities. Children completed the PROMIS profile-25, Kidscreen-10 index, DISABKIDS-37 and other instruments. We calculated a utility score based on Kidscreen and the algorithm by Chen et al. (2014) [1] creating a Kidscreen utility score (KUS). We investigated internal consistency of the KUS, as well as convergent validity. The utility algorithm used a model based on MM estimate with six stepwise-selected Kidscreen items (Healthy, Energy, Sad, Lonely, School, Alert). A linear regression analysis was used to explain the variance in PROMIS profile scores.

**Results:** The study included 33 children receiving standard care for type 1 diabetes who completed the self-report instruments. Age range was 8–18 years (mean 13 years). The KUS ranged from 0.64 to 1.06 with a mean of 0.91. Having acceptable internal consistency (Chronbach’s alpha 0.809) it correlated strongly with DISABKIDS r(24) = 0.582, p < 0.002 and with the PROMIS profile scores (r = 0.378 to r = 0.596). A linear regression using the KUS as the independent variable explained a modest proportion of the variance of the PROMIS scores: Anxiety R^2^ = 0.074, Depressive symptoms R^2^ = 0.049, Physical function R^2^ = 0.160, Fatigue R^2^ = 0.144, Peer relationships R^2^ = 0.050, Pain interference R^2^ = 0.105.

**Conclusions:** This study explored the relationship between paediatric PROMIS-25 and a utility measure. A limitation of the study is the size of the population; however, the data collection provided the possibility of a preliminary investigation. Further exploration of the relationship between PROMIS-25 and utility measurements would benefit from a larger population and the calibration of the utility weights generated by different utility instruments.

References

[1] Chen C., Stevens S., Rowen D., Ratcliffe J. From KIDSCREEN-10 to CHU9D: creating a unique mapping algorithm for application in economic evaluation. Health and Quality of Life Outcomes 2014, 12:134.

### O71 Implementation of PROMIS scales in a cerebrovascular clinic—experience after 10 years

#### Irene Katzan, Brittany Lapin, Nicolas Thompson, Andrew Russman, Ken Uchino

##### Cleveland Clinic, Cleveland, OH, USA

###### Correspondence: Irene Katzan

katzani@gmail.com

*Journal of Patient-Reported Outcomes* 5(1): O71.

**Objective:** Stroke patients often have “hidden deficits” that impair their health-related quality of life (hrQoL) such as fatigue, cognitive symptoms, and depression and which are best measured using patient self-report. To better understand and optimize outcomes of stroke survivors, the Cleveland Clinic Cerebrovascular Center began collection of patient-reported outcomes (PROs) within the ambulatory clinics in late 2008. We describe our experience with collection and clinical utilization of PROs in the cerebrovascular clinics of a large healthcare system.

**Methods:** Implementation occurred as part of a larger patient-entered data initiative within Cleveland Clinic. PROs were initially collected through an internally developed patient data collection platform and were migrated to Epic tools in November 2019. Patient questionnaires included PHQ-9 depression screen, a sleep apnea scale, PROMIS Global Health and computer adaptive testing versions of PROMIS physical function, fatigue, pain interference, sleep disturbance, satisfaction with social roles and NeuroQoL cognitive function. Clinicians also record clinical information regarding patients’ cerebrovascular disease in structured fields within the EHR. T-scores can be viewed graphically over time in Epic’s Synopsis reports. Score percentiles are automatically inserted into documentation templates.

**Results:** Since starting data collection in 2008, PROs have been collected in 39,863 visits, representing 22,542 unique patients. Completion rates have consistently been over 50%. Patients who complete questionnaires are younger (58.7 [SD 15.8] vs. 62.0 [SD 15.8], P < 0.001) and have lower clinician-reported disability scores (mean modified Rankin scale 1.13 [SD 1.13] vs. 1.39 [SD 1.26], P < 0.001). The majority (58.9%) of patients have at least one score ≥ 1 SD worse than the US population mean and 38.3% have 2 + scores *besides PROMIS physical function* that are ≥ 1 SD worse than the population mean. There is wide variability in severity of symptoms among patients with similar clinician-reported disability and neurological deficits. We will provide examples along with actions that can be taken based on PROMIS scores.

**Conclusions:** PRO collection in a cerebrovascular clinic is feasible. They have dramatically improved our understanding of the health status of our stroke patients and has informed clinical management. Development of evidence-based interventions for PRO scores will further improve their usefulness in ambulatory stroke care.

### O72 Patient factors associated with improvements in PROMIS Physical Function and Pain Interference after spine surgery

#### Jacquelyn Pennings, Rogelio Coronado, Hiral Master, Kristin Archer

##### Vanderbilt University Medical Center, Nashville, TN, USA

###### Correspondence: Jacquelyn Pennings

jacquelyn.pennings@vumc.org

*Journal of Patient-Reported Outcomes* 5(1): O72.

**Objective:** To assess demographic and clinical/surgical characteristics associated with improvement in PROMIS physical function, and pain interference and contrast them with predictors of the typical legacy measures of ODI, NDI, and NRS pain intensity after spine surgery for degenerative conditions.

**Methods:** 727 degenerative lumbar and cervical spine surgery patients with preoperative and 12-month follow-up PROMIS data who underwent spine surgery at a single institution were analyzed. Demographic (age, gender, race, smoking status, education, insurance, liability claim, employment status), clinical/surgical characteristics (preop opioid use, comorbidities, procedure, revision status), and preoperative outcome scores were entered as predictors of 12-month PROMIS (PF and PI) and legacy measures (ODI/NDI, NRS axial pain, NRS extremity pain). The 4-item PROMIS short forms were used to assess PF and PI. Predictor importance, coefficients, and overall model R^2^ values are presented.

**Results:** As expected, the baseline scores associated with each outcome had the highest predictor importance. Other predictors that were significant for both 12-month PF (R^2^ = 0.38) and ODI/NDI (R^2^ = 0.37) included preop opioid use, preop PROMIS depression, employment status, comorbidity count, and education. BMI, smoking status, and age were only significant predictors of PROMIS PF while race, revision status, and pain intensity were only significant for ODI/NDI. Procedure, liability status, lumbar vs cervical, gender, and insurance status were not significant predictors of either outcome in these models. Significant predictors of PROMIS PI (R^2^ = 0.28) were preop score, preop opioid use, employment, education, PROMIS preop depression, smoking status, comorbidities, and race. Significant predictors of NRS pain scores were similar with a few differences.

**Conclusions:** Previous research shows PROMIS measures are reliable, valid, and responsive in spine surgery patients. As PROMIS measures are now being used to evaluate surgical outcomes more frequently through their incorporation into standard hospital data collection, registries, and trials, it is important to understand the patient demographic and clinical/surgical characteristics that are associated with PROMIS outcomes after spine surgery. Predictors of PROMIS and legacy measures were similar but not identical. Contrasting these predictors of PROMIS outcomes with legacy measures aids surgeons and researchers in understanding how these PROMIS measures are similar but distinct from legacy outcomes.

### P73 Influence of goal attainment scaling on cognitive-behavioral based physical therapy outcomes after lumbar spine surgery

#### Rogelio Coronado^1^, Hiral Master^1^, Jordan Bley^1^, Payton Robinette^1^, Michael O'Brien^1^, Emma Sterling^1^, Abigail Henry^1^, Jacquelyn Pennings^1^, Susan Vanston^1^, Brittany Myczkowski^1^, Richard Skolasky^2^, Steven Wegener^2^, Kristin Archer^1^

##### ^1^Vanderbilt University Medical Center, Nashville, TN, USA. ^2^Johns Hopkins Medicine, Baltimore, MD, USA

###### Correspondence: Jacquelyn Pennings

jacquelyn.pennings@vumc.org

*Journal of Patient-Reported Outcomes* 5(1): P73.

**Objective:** Patient-centered goal setting is an important component of behavioral interventions for chronic pain. Limited data exist on the relationship between goal attainment during cognitive-behavioral based physical therapy (CBPT) and pain-related outcomes. The objective was to examine the relationship between CBPT goal attainment and pain-related outcomes in a cohort of patients who completed a 6-week telephone-based CBPT intervention after lumbar spine surgery.

**Methods:** Secondary analysis from a randomized trial comparing postoperative CBPT and an education program was used for this study. 108 participants (mean 63.5 ± 11.3 years) who completed the CBPT intervention were included. Six and 12-month outcomes included physical function (PROMIS), pain interference (PROMIS), and back and leg pain intensity (Brief Pain Inventory). At each session, CBPT participants used Goal Attainment Scaling (GAS) to set goals and report goal attainment from the previous session. The number, type, and percentage of goals met was recorded. A GAS t-score for achievement of goals across all sessions was computed for each patient. Patients were grouped as high or low goal attainment based on a GAS t-score of 50 (goals met as expected). Outcome differences between groups at each follow-up time point were examined using linear regression controlling for baseline outcome score.

**Results:** Participants set a median of 3 goals (range: 1–6) at each CBPT session. The most common goals were related to participating in physical/recreational activity (36%), adopting a cognitive or behavioral strategy (28%), exercising (11%), or performing activities of daily living (11%). The least common goals related to social activities (2%). 43% participants met criteria for high goal attainment (mean % goals met = 87% compared to 64% in the low goal attainment group, p < 0.001). Greater improvements in the high goal attainment group were observed in PROMIS physical function at 6 (beta = 4.2, p < 0.05) and 12 months (beta = 3.5, p < 0.05) and back pain intensity at 12 months (beta = − 0.9 p < 0.05).

**Conclusions:** The attainment of personalized goals within a CBPT intervention was an important factor related to improvements in physical functioning after spine surgery. Implementing a structured process for setting personalized goals and tracking goal attainment may be an essential aspect of a biopsychosocial approach for addressing functional impairment following surgery.

### P74 Trends in PROMS-29 throughout COVID-19 in the Canadian Early Arthritis Cohort (CATCH)

#### Susan Bartlett^1,2^, Orit Schieir^3^, Marie-France Valois^4^, Vivian Bykerk^5^

##### ^1^McGill University, Montreal, Canada. ^2^Johns Hopkins Medicine, Baltimore, D, MUSA. ^3^University of Toronto, Toronto, Canada. ^4^McGill, Montreal, Canada. ^5^Hospital for Special Surgery, New York, NY, USA

###### Correspondence: Susan Bartlett

susan.bartlett@mcgill.ca.

*Journal of Patient-Reported Outcomes* 5(1): P74.

**Objective:** With the COVID-19 pandemic, adults with rheumatoid arthritis faced increased risks of infection and hospitalization and potentially greater social restrictions due to their use of potent immunosuppressant medications. We examined trends in PROMIS-29 domains prior to and during the first year of the COVID-19 pandemic in adults with RA.

**Methods:** Data were from RA patients enrolled in the Canadian Early Arthritis Cohort who had completed PROMIS-29 at study visits between 9/2019 and 1/2021. Descriptive statistics were calculated to evaluate changes in PROMIS-29 domains 6 months prior to, and during Wave 1 (March to June 2020) and Wave 2 (October to Jan 2021).

**Results:** Participants (N = 468) were mostly white (89%) women (70%) with a mean (SD) age of 60 (15) years. Prior to the pandemic, mean PROMIS-29 scores were in the normal range, with the lowest being Physical Function (46.7) and the highest Pain Interference (52.3). Analyses of monthly trends showed that HRQL impacts were greatest in April 2020. Average PROMIS-29 scores worsened for all domains except Participation (mean change − 0.2). The largest changes were in Depression (+ 4.8) and Anxiety (+ 4.3). As compared with Jan 2020, in April higher proportions of participants reported moderate-severe anxiety (28% vs 40%), depression (18% vs. 34%), fatigue (22% vs. 40%), sleep disturbance (18% vs. 34%), pain (23% vs. 38%), and disability (21% vs 34%).

By July 2020, mean PROMIS-29 scores had decreased in depression (− 4.4), anxiety (− 3.2), fatigue (− 1.6) and participation (− 1.4). The proportions of patients reporting moderate-severe symptoms (e.g., ≥ 60) were similar to before the pandemic for all domains except physical function where 29% continued to report moderate-severe impairments. In Wave 2, scores were higher than pre-pandemic levels, but lower than Wave 1, with the largest changes in depression and anxiety.

**Conclusions:** Modest impacts over the first year of variable COVID-19 pandemic restrictions were observed on HRQL of adults with RA in Canada. The largest changes were in anxiety and depression during both Waves. Greater impairments in physical function have persisted up to 12 months into the pandemic.

### O75 Many better, many worse: mean PROMIS-29 Scores mask significant shifts during COVID-19 in RA

#### Susan Bartlett^1,2^, Dana DiRenzo^2^, Michelle Jones^2^, Clifton Bingham^2^

##### ^1^McGill University, Montreal, Canada. ^2^Johns Hopkins Medicine, Baltimore, MD, USA

###### Correspondence: Clifton Bingham

cbingha2@jhmi.edu

*Journal of Patient-Reported Outcomes* 5(1): O75.

**Objective:** Rheumatic diseases (RD) are chronic conditions that require potent immunosuppressants to control systemic inflammation. Fears associated with increased vulnerability from being on immunosuppressants plus medications shortages (e.g., hydroxychloroquine) resulted in considerable stress for patients in the early months of the COVID-19 pandemic. We evaluated changes in HRQL in the initial months of the COVID-19 in adults with RD and hypothesized that multiple PROMIS-29 domains scores would be negatively impacted.

**Methods:** The sample included patients followed (virtually or in-person) in Rheumatic Disease clinics at Johns Hopkins 3/15/2020 to 6/30/2020. Patients complete the PROMIS-29 as part of routine care, and scores were compared with the most recent visit prior to 3/15. Anxiety was classified as worse (≥ 4.0 points), same (− 3.9 to 3.9) or better (≤ − 4.0) at the second visit.

**Results:** Data were available for 151 patients with a mean (SD) age of 55 who were mostly white (81%) women (73%) with RA (50%), PSA (27%), AS/SPA (15%) or other RD (9%). Mean (SD) changes in PROMIS-29 scores ranged from − 0.9 for Fatigue [7.6] and Depression [7.9] to 1.4 (9.7) for Anxiety. 45 (30%) patients were classified with worse anxiety, 40 (27%) with improved anxiety, and 66 (44%) the same. Change in anxiety was not associated with age, sex, race, or disease.

Among patients reporting worse anxiety (mean [SD] change + 12.3 [9.0]), Depression was significantly worse (3.2 [8.0]) compared to patients with same (− 1.2 [6.9] or improved anxiety (− 5.0 [7.0]) (p’s < 0.05). Among patients whose anxiety improved, Fatigue (− 4.4 [7.9]) and Participation (3.1 [7.8] improved. Changes in anxiety were not associated with changes in physical function.

Patient Global Impression of Disease Change scores (N = 128) indicated 34% of patient reported their disease was worse, 30% had improved, and 36% were the same. Most (88%) patients with worse anxiety reported worse (45%) or the same (43%) disease activity; 43% with improved anxiety had improved disease, 30% the same, and 27% were worse.

**Conclusions:** While the average within-person change in PROMIS-29 scores were trivial, a substantial proportion of patients experienced worsening or improved anxiety which also tracked with meaningful changes in several other PROMIS-29 domains.

### P76 An investigation of the physical disability experienced by people with Multiple Sclerosis

#### Paul Kamudoni^1^, Jeffrey Johns^2^, Dagmar Amtmann^3^, Karon Cook^4^, Rana Salem^3^, Sam Salek^5^, Jana Raab^1^, Rod Middleton^6^, Christian Henke^1^

##### ^1^Global Evidence & Value Development-R&D, Merck Healthcare KgaA, Darmstadt, Germany. ^2^Institute of Medicines Development, Cardiff, United Kingdom. ^3^Department of Rehabilitation Medicine, University of Washington, Seattle, WA, USA. ^4^Feral Scholars, Broaddus, TX, USA. ^5^School of Life and Medical Sciences, Hartfield, United Kingdom. ^6^UK MS Register, Swansea Medical School, Swansea, United Kingdom

###### Correspondence: Paul Kamudoni

pkamudoni@gmail.com.

*Journal of Patient-Reported Outcomes* 5(1): P76.

**Objective:** To evaluate patient-reported physical disability, based on physical function, walking ability and upper extremity (UE) function assessments, among Multiple Sclerosis (MS) patients with different disease characteristics, and to explore the feasibility of assessing the various domains.

**Methods:** Data were obtained from a 96-week longitudinal study carried out in the UK MS Register Cohort, from September 2018 to October 2020. Target outcome measures included the MSWS-12, the NeuroQoL Upper Extremity Short form, and the PROMISnq Physical Function-MS 15a. In addition, the PROMIS Physical Function-CAT was also analyzed. Analyses performed included summary descriptive statistics, ANOVA test for group differences, spearman’s correlation analysis, and item utilization rates for the CAT.

**Results:** In total, 284 patients were included in the current analyses (76% female; mean age, 50.9 ± 9.4). Mean ± SD scores on the MSWS-12, the NeuroQoL UE-SF, and the PROMISnq PF-MS 15a were 36.8 ± 27.02, 43 ± 9.6, and 40.7 ± 9.8, respectively. The largest proportion of the sample required no assistance with mobility (MSWS-12, scores 0 to 20; 37%), and had mild limitations or problems with upper extremity function (NeuroQoL UE-SF, T-scores 31 to 46; 50.4%), or physical function (PROMISnq PFMS 15a, T-scores 26 to 50; 47.5%). The proportion of patients with the highest/lowest possible score were 12%/2.11% (MSWS-12), 0.35%/38.4%, and 0.35%/4.6%, respectively. All three target measures showed expected differences across distinct disability patient groups. For the PROMIS PF-CAT, items related to upper extremity were least frequently selected (2 items of all 20 items administered) than mobility or activities of daily living questions, and at high disability levels only.

**Conclusions:** Impairments in physical function and upper extremity function were mild-to-moderate, whilst mobility was assistance-free or with gait problems (i.e., mild or no limitations), for most of our sample. Unlike mobility (MSWS-12) or physical function (PROMISnq PF-MS 15a), the measurement features of upper extremity function (NeuroQoL UE-SF) e.g., ceiling effects, suggested limited measurement feasibility in the current study population.

### O77 Neighborhood environment and health related quality of life for children with chronic conditions

#### Jin-Shei Lai^1^, Katy Wortman^1^, Amy Paller^1^, Stewart Goldman^2^

##### ^1^Northwestern University, Chicago, IL, USA. ^2^Phoenix Children’s Hospital & the University of Arizona College of Medicine Phoenix, Phoenix, AZ, USA

###### Correspondence: Jin-Shei Lai

js-lai@northwestern.edu

*Journal of Patient-Reported Outcomes* 5(1): O77.

**Objective:** Evidence has indicated that the social and physical neighborhood environment exerts significant effects on health and health-related quality of life. However, such research on children with chronic conditions was lacking. This study aims to evaluate whether social-economic (deprivation index and socio-economic status) and physical (walkability) attributes of the neighborhood environment are associated with health-related quality of life for children with brain tumors (BT) or atopic dermatitis (AD).

**Methods:** Data from 155 children with AD (mean age = 11.9 yrs) and 73 children with BT (mean age = 14.5 years) were analyzed. Children were recruited during their clinical visits and completed PROMIS Anxiety, Depression, Fatigue, Mobility, and Peer Relationship. Residential addresses of study participants were geocoded and assigned to community deprivation index by census block group using Decentralized Geomarker Assessment for Multi-Site Studies (DeGAUSS) technology. Median value of owner-occupied housing within block group represented neighborhood socio-economic status (SES). The national walkability index (walkability) was obtained using a nationwide geographic data resource that ranks block groups according to their relative walkability. PROMIS measures were reported on the T-score (mean = 50, SD = 10) metric. Multivariable regression models were used, with outcome variables: PROMIS Anxiety, Depression, Fatigue, Mobility, and Peer Relationship; and independent variables: deprivation index, walkability, SES, diagnosis (BT, AD), gender, and age.

**Results:** Deprivation index was significantly (p < 0.05) associated with Fatigue and Peer Relationship; in which more disadvantaged children (i.e., higher Deprivation index) reported worse fatigue and peer relationships. Gender was significantly (p < 0.05) associated with Anxiety and Depression; in which female children reported more anxiety and depression than male children. Disease was significantly associated with Mobility and Peer Relationships; in which children with AD reported better mobility and peer relationships than children with BT. Neighborhood SES, walkability, and age were not significantly associated with any PROMIS measures.

**Conclusions:** This study explored association of social and physical environmental factors with health-related quality of life in children with chronic conditions. Results showed deprivation index was associated with PROMIS scores but not neighborhood SES and walkability. Future studies should be conducted to evaluate whether the same conclusions can be made on children with other conditions.

### P78 PROMIS Mental Health and Physical Health summary scores in patients on kidney replacement therapies

#### Istvan Mucsi^1^, Gaauree Chala^1^, Anqi Chen^1^, Nathaniel Edwards^1^, John D. Peipert^2^, Susan J. Bartlett^3^, Doris Howell^4^, Madeline Li^5^, Ron D. Hays^6^

##### ^1^Ajmera Transplant Program, University Health Network, Toronto, Canada. ^2^Feinberg School of Medicine, Northwestern University, Chicago, IL, USA. ^3^3Center for Health Outcomes Research, McGill University, Montreal, Canada. ^4^Lawrence S. Bloomberg Faculty of Nursing, University of Toronto, Toronto, Canada. ^5^Princess Margaret Cancer Center, Toronto, Canada. ^6^University of California, Los Angeles, Los Angeles, CA, USA

###### Correspondence: Istvan Mucsi

istvan.mucsi@utoronto.ca.

*Journal of Patient-Reported Outcomes* 5(1): P78.

**Objective:** PROMIS profiles include multiple health-related quality of life domains and higher-order summary scores. We assessed the construct validity of the PROMIS physical (PH) and mental health (MH) summary scores among patients treated with dialysis or kidney transplant.

**Methods:** A cross-sectional convenience sample of adults treated with kidney transplant or dialysis completed the PROMIS 29 2.0 or CATs using electronic data capture. Participants also completed a sociodemographic questionnaire and “legacy” questionnaires (Patient Health Questionnaire-9 [PHQ9], Medical Outcomes Study Short Form-12 [SF-12] and the EQ-5D-5L). The SF6D utility score was calculated from SF-12 item scores. Clinical data were extracted from medical records. Convergent validity was assessed using Pearson correlation between MH score vs PHQ9 and SF12 mental component summary (MCS); between PH score vs serum albumin and SF12 physical component summary (PCS). We assessed correlations between MH and PH summary scores and EQ5D-5L and SF-6D health utility scores. Furthermore, we compared the summary scores between groups expected to have different PH and MH based on prior literature or clinical knowledge: dialysis vs kidney transplant; anemia vs no anemia (Hb 125); high vs low comorbidity (Charlson comorbidity index [CCI] 3); no/mild vs moderate/severe depressive symptoms (PHQ9 10).

**Results:** Of the 602 participants, mean (SD) age was 58(17) years, 59% male and 45% White; 48% had received a kidney transplant. The mean(SD) PH and MH T-score was 42(11) and 49(10), respectively. Our analysis yielded correlations in the expected directions. For PH: PCS(r = 0.81), serum albumin (r = 0.37); for MH: PHQ9(r = − 0.74) and MCS(r = 0.65). Both PH and MH scores correlated with the EQ5D-5L (r = 0.65 and 0.61, respectively) and with the SF-6D (0.78 and 0.79, respectively). Both PH and MH scores were higher among kidney transplant recipients than patients on dialysis (48(10) vs 37(9); 51(9) vs 47(10), respectively; p < 0.001). The expected differences (> 5 point for each comparisons) were seen for the additional “known group” comparisons.

**Conclusions:** These results support the validity of PROMIS PH and MH scores among patients treated with kidney replacement therapies. These summary scores may be useful to monitor health-related quality of life in research and clinical settings.

